# Supplementary figures and images for: Metabolic requirement for GOT2 in pancreatic cancer depends on environmental context
Source: eLife. 2022 Jul 11;11:e73245. doi: 10.7554/eLife.73245 (PMC9328765; doi:10.7554/eLife.73245)

**Figure 1B**

Blot1-GOT2


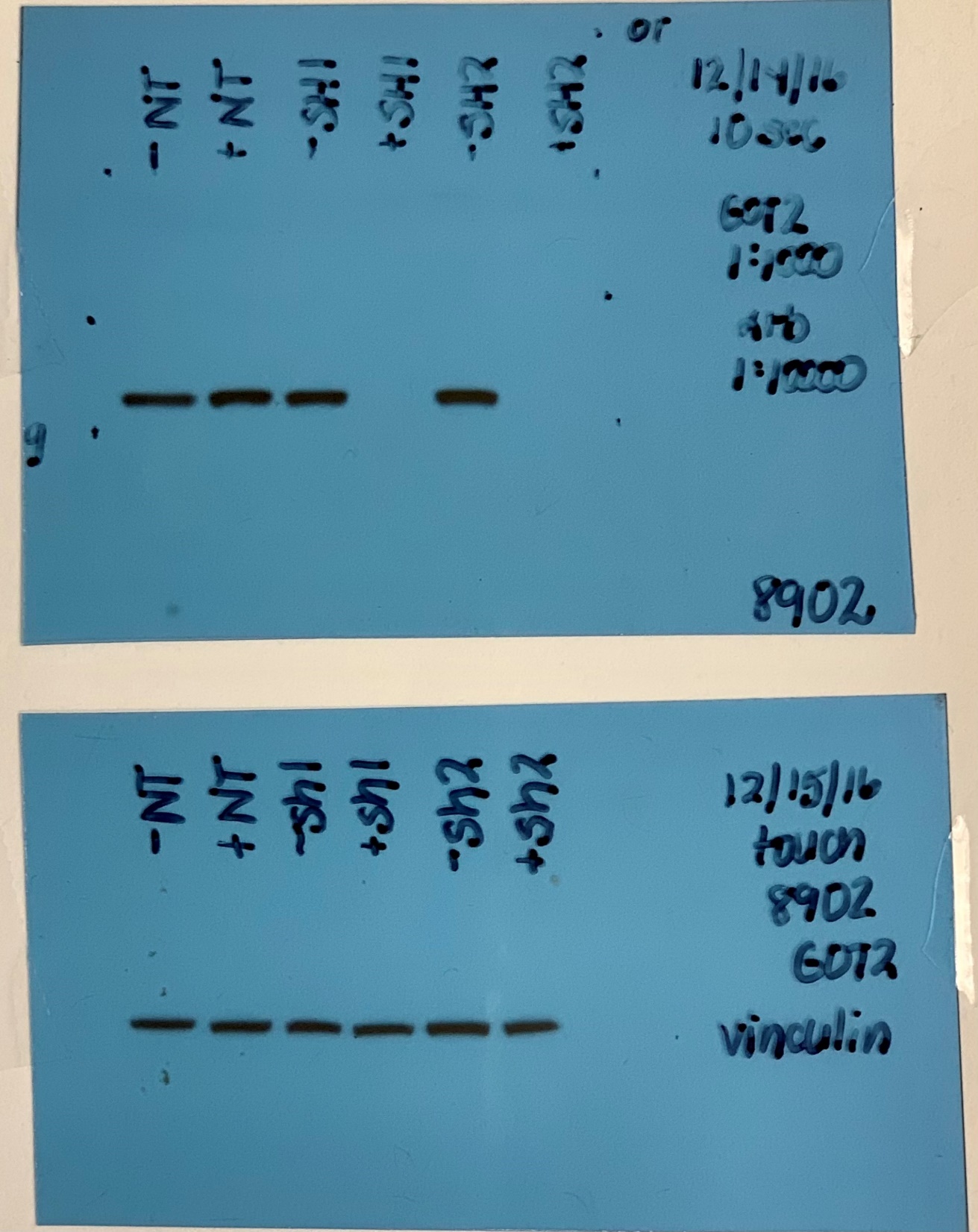


Blot2-Vinculin loading control


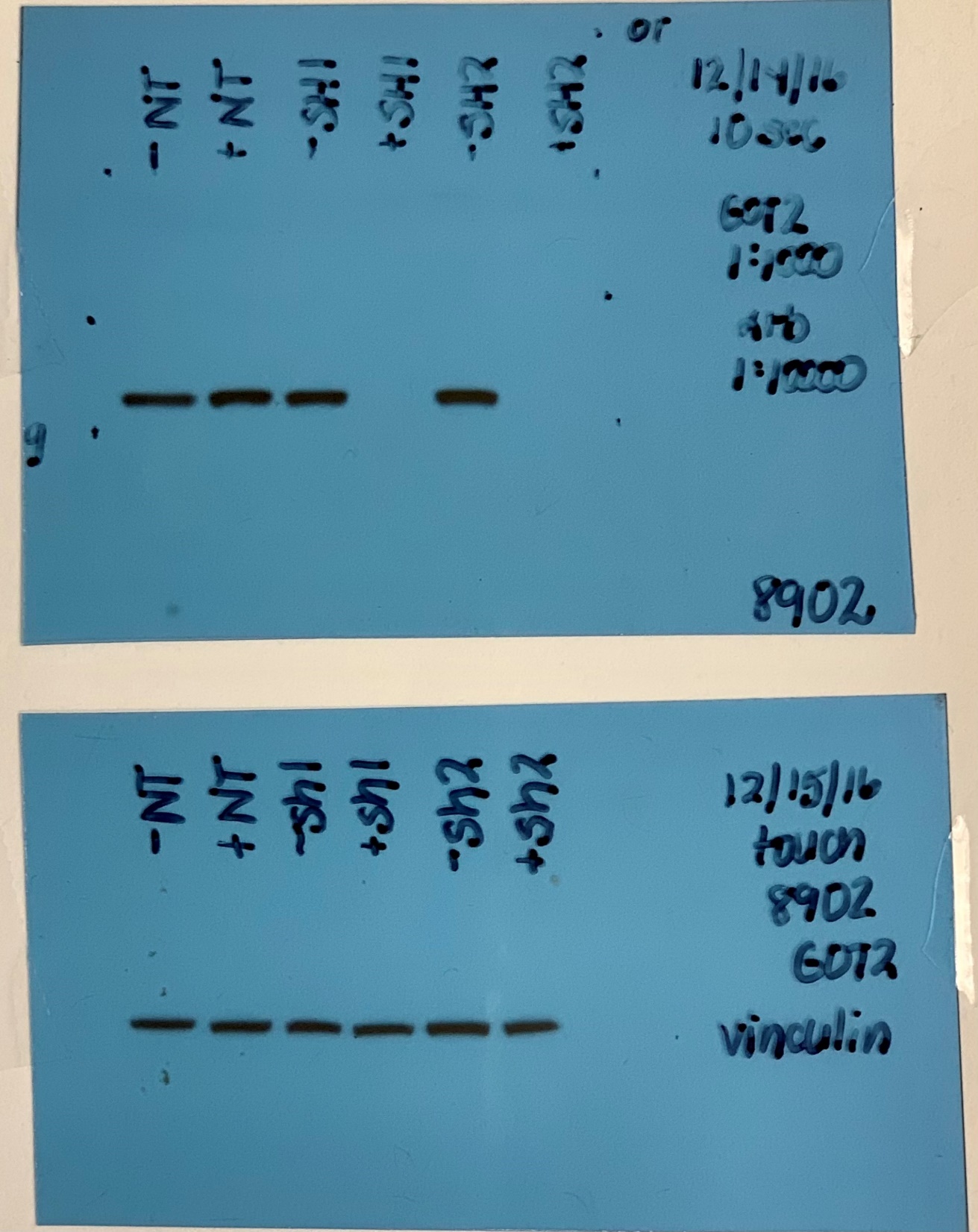

Supplement: Figure 1—source data 1. [file elife-73245-fig1-data1.zip › Figure 1-source data 1.docx]

**Figure 2-figure supplement 1D**


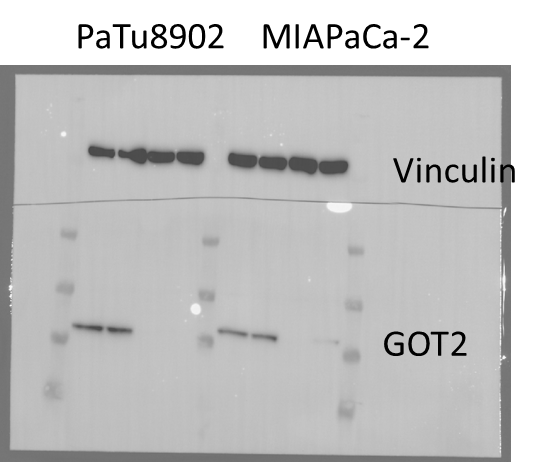

Supplement: Figure 2—figure supplement 1—source data 1. [file elife-73245-fig2-figsupp1-data1.zip › Figure 2-figure supplement 1-source data 1.docx]

**Figure 2-figure supplement 2A**


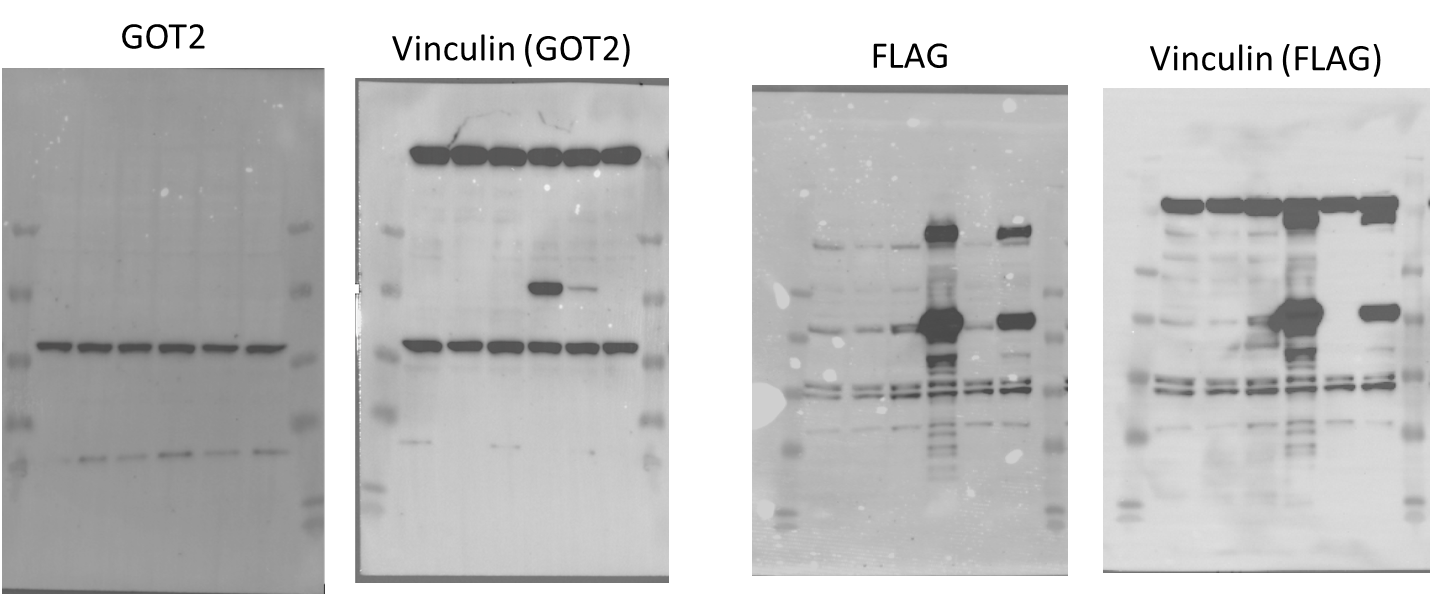
PaTu-8902 shNT


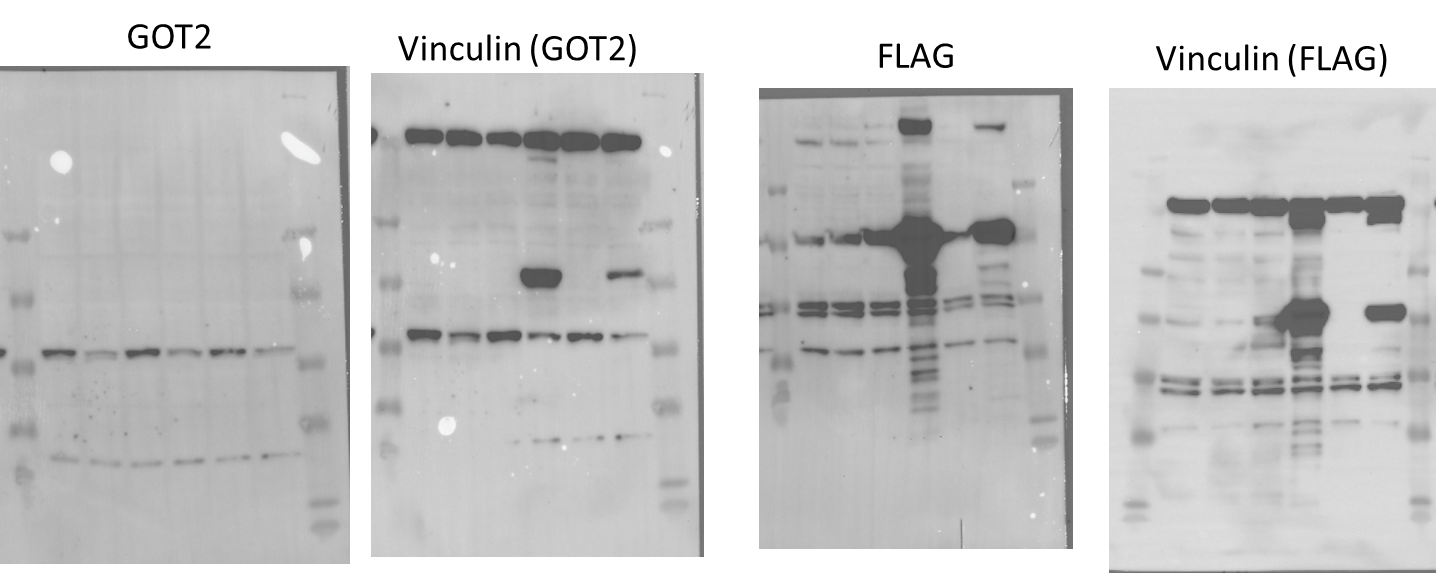
PaTu-8902 shGOT2.1

MIAPaCa-2 shNT & shGOT2.1


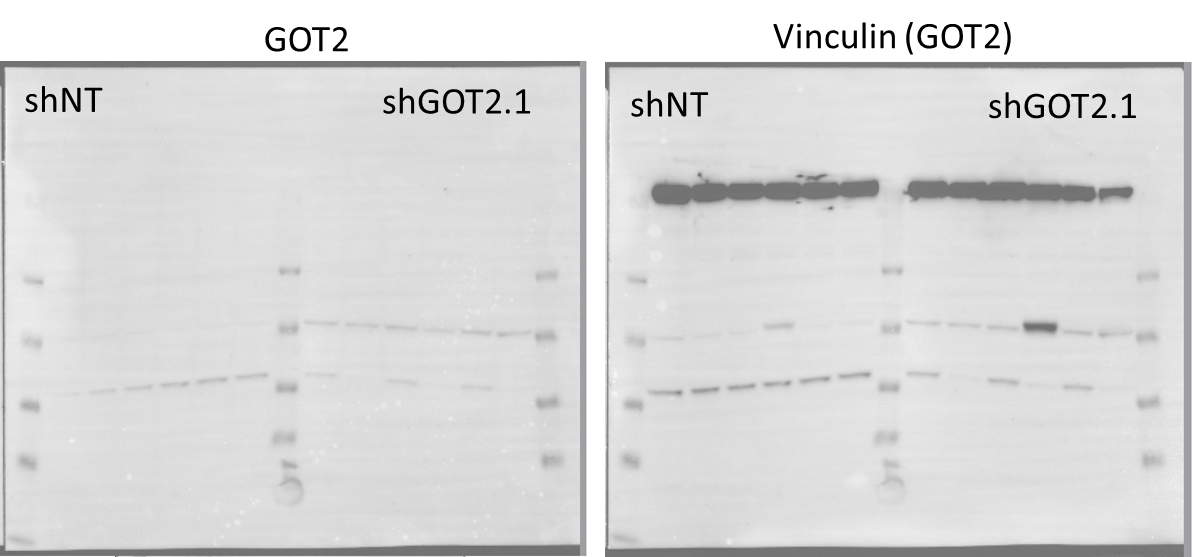

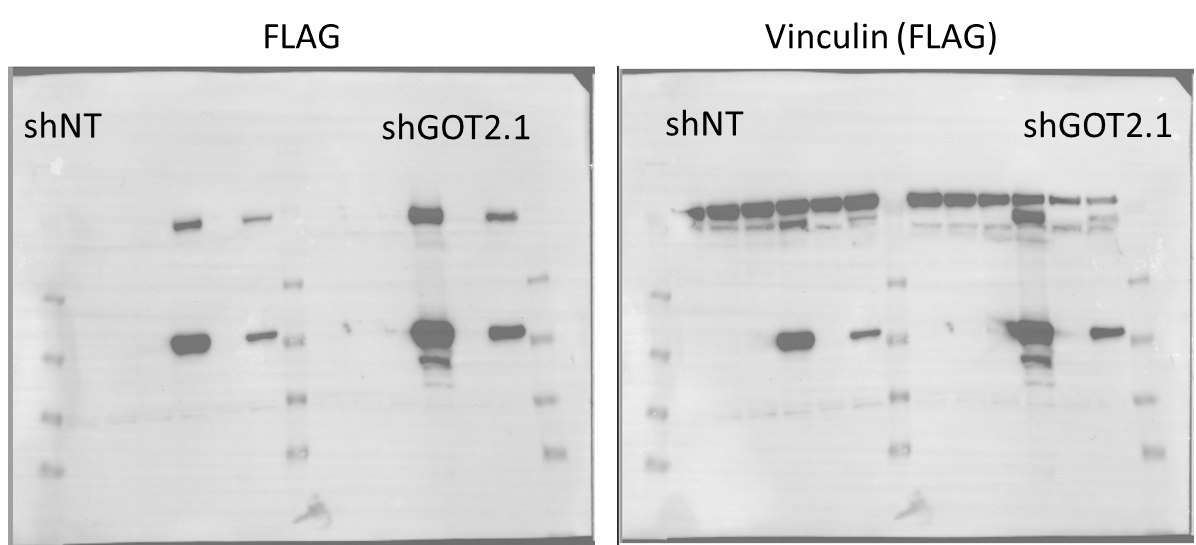

Supplement: Figure 2—figure supplement 2—source data 1. [file elife-73245-fig2-figsupp2-data1.zip › Figure 2-figure supplement 2-source data 1.docx]

**Figure 6E**

Blot1-GOT2, MCT1, Vinculin loading control


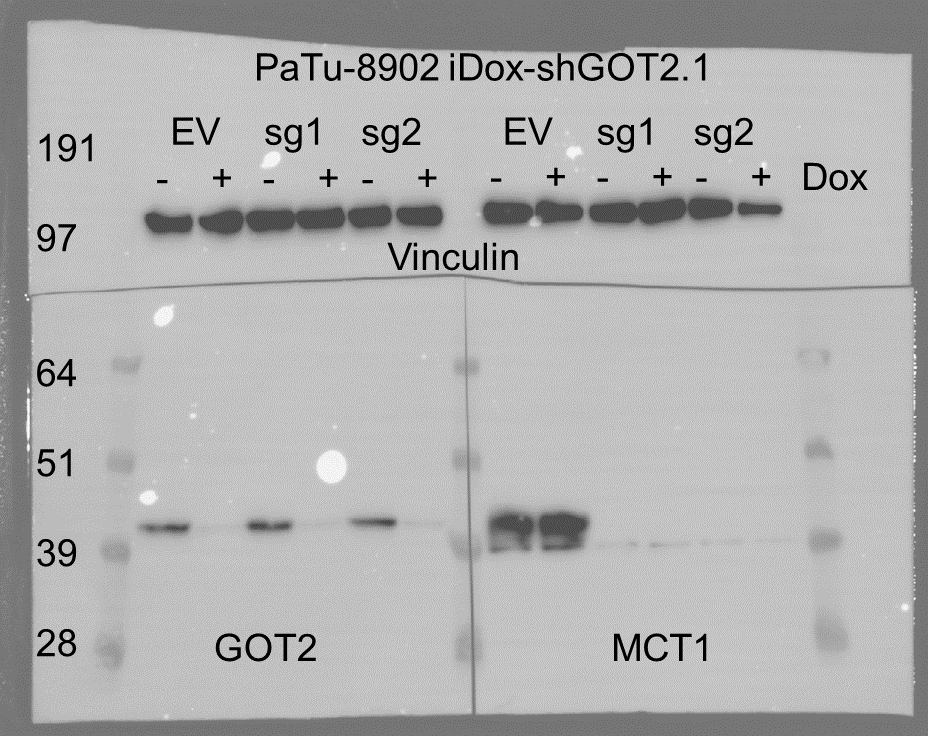

Supplement: Figure 6—source data 1. [file elife-73245-fig6-data1.zip › Figure 6-source data 1.docx]

**Figure 6-figure supplement 1B**


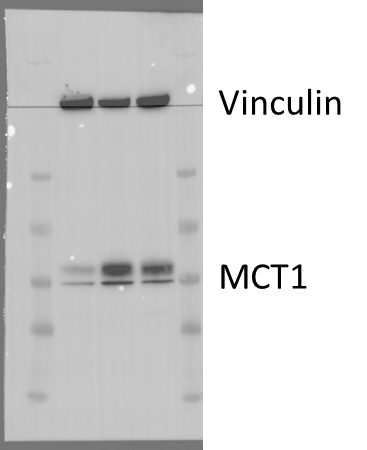


**Figure 6-figure supplement 1D & 1F**

**
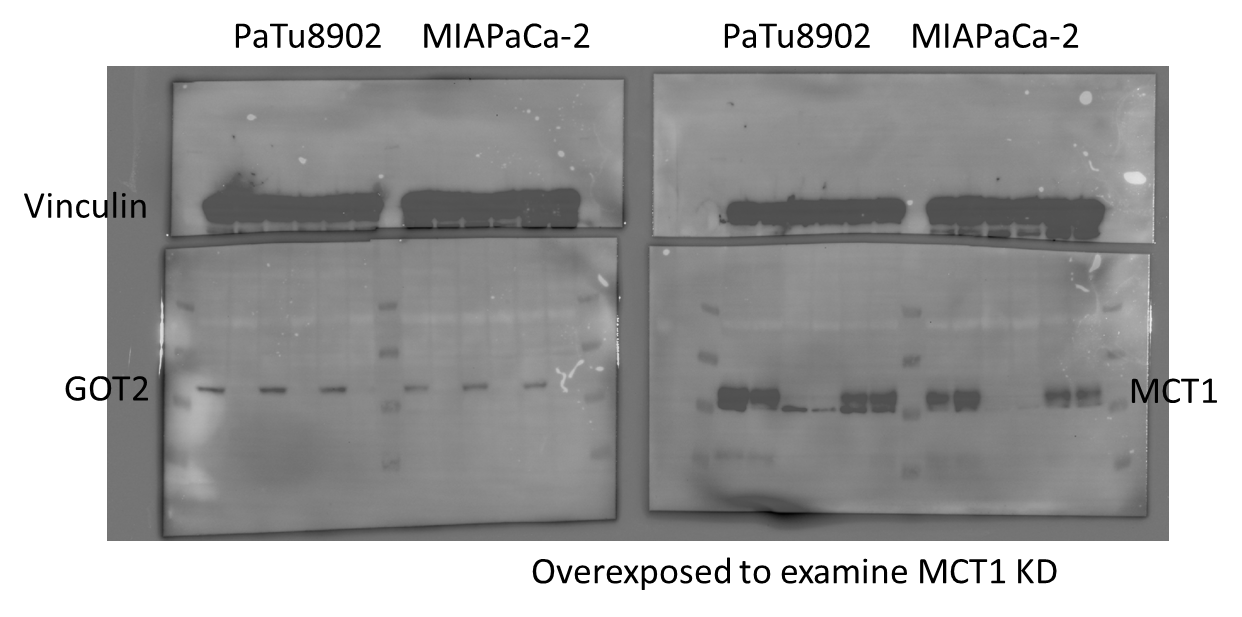
**

**Figure 6-figure supplement 1J**


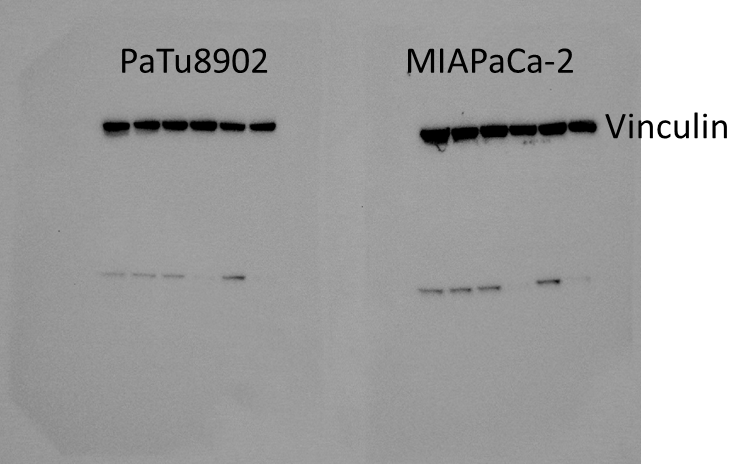


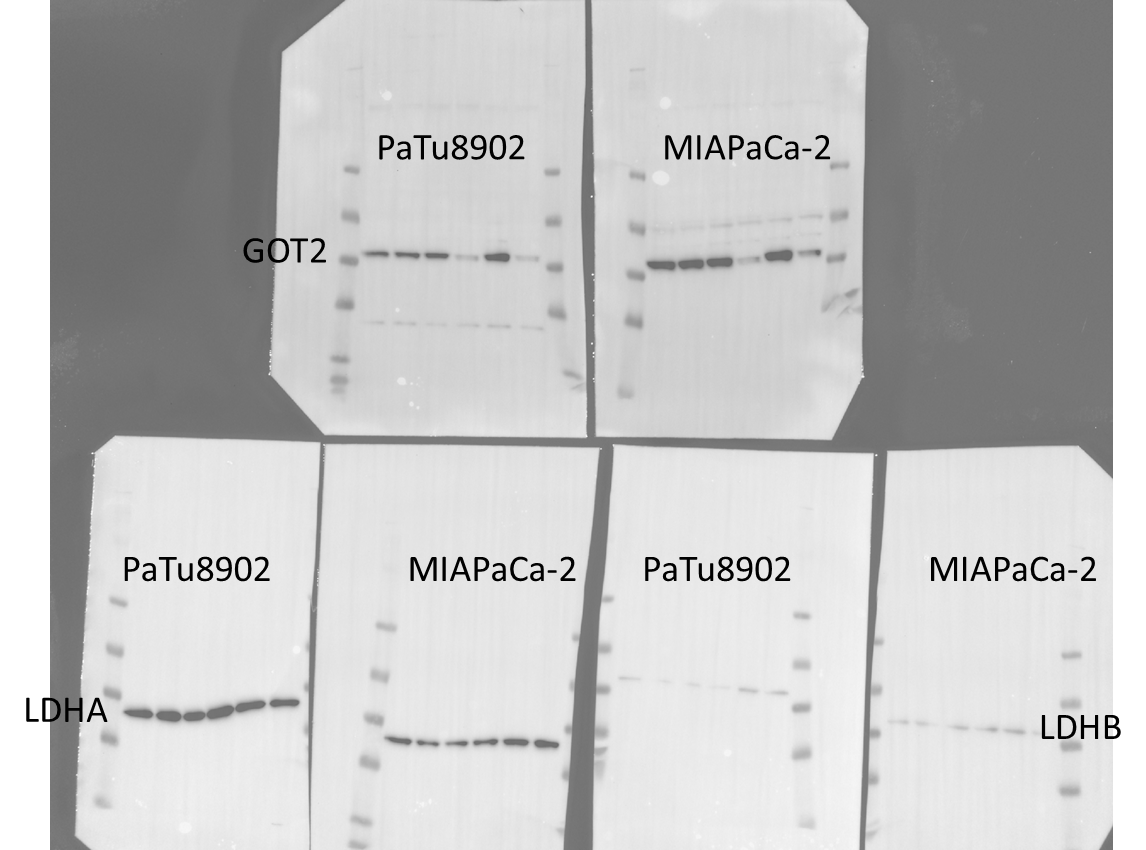

Supplement: Figure 6—figure supplement 1—source data 1. [file elife-73245-fig6-figsupp1-data1.zip › Figure 6-figure supplement 1-source data 1.docx]
