# Supplementary material for: Metabolic requirement for GOT2 in pancreatic cancer depends on environmental context
Source: eLife. 2022 Jul 11;11:e73245. doi: 10.7554/eLife.73245 (PMC9328765; doi:10.7554/eLife.73245)

**Figure 1-figure supplement 1A**

MIAPaCa-2


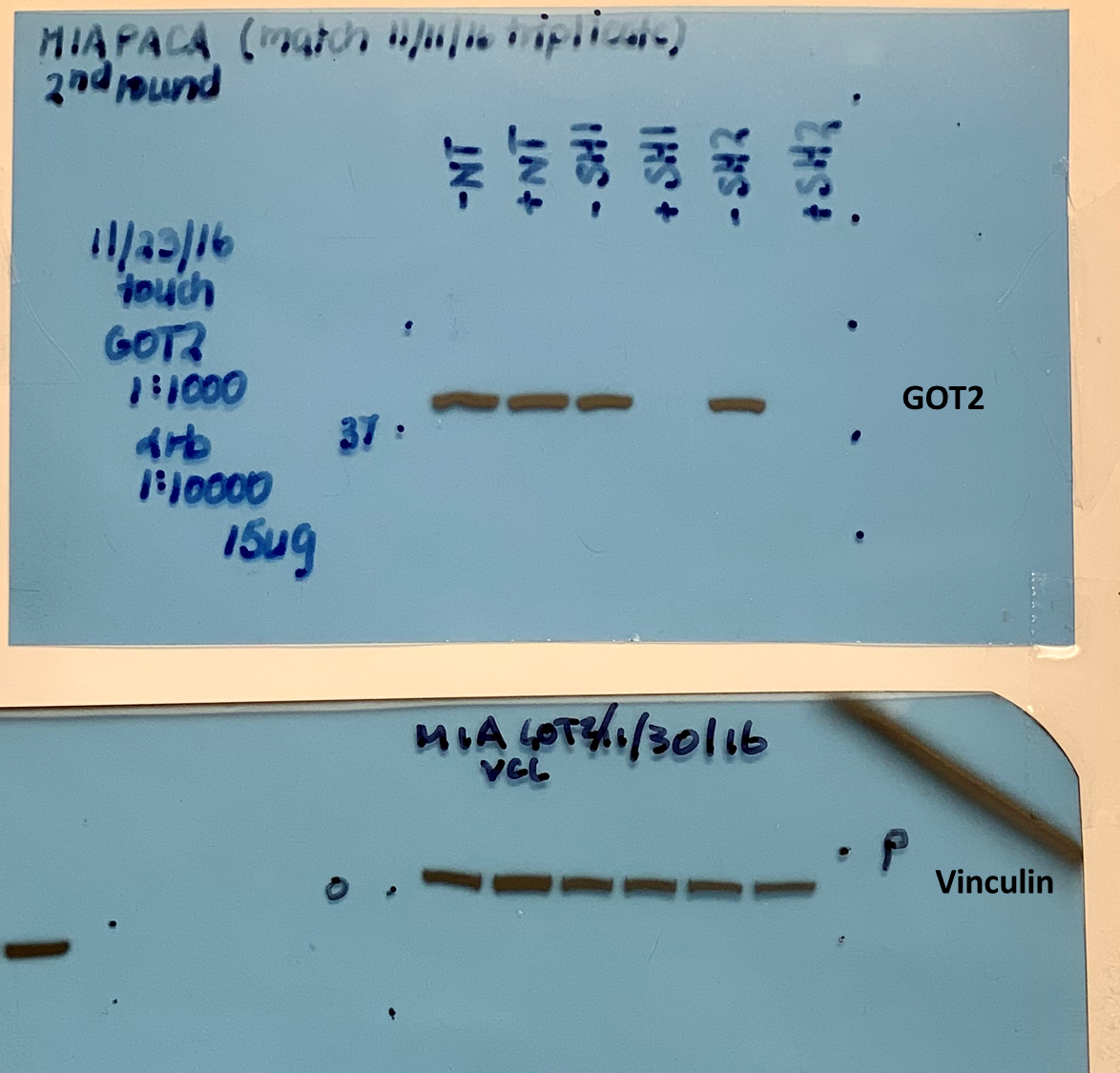


Capan-1


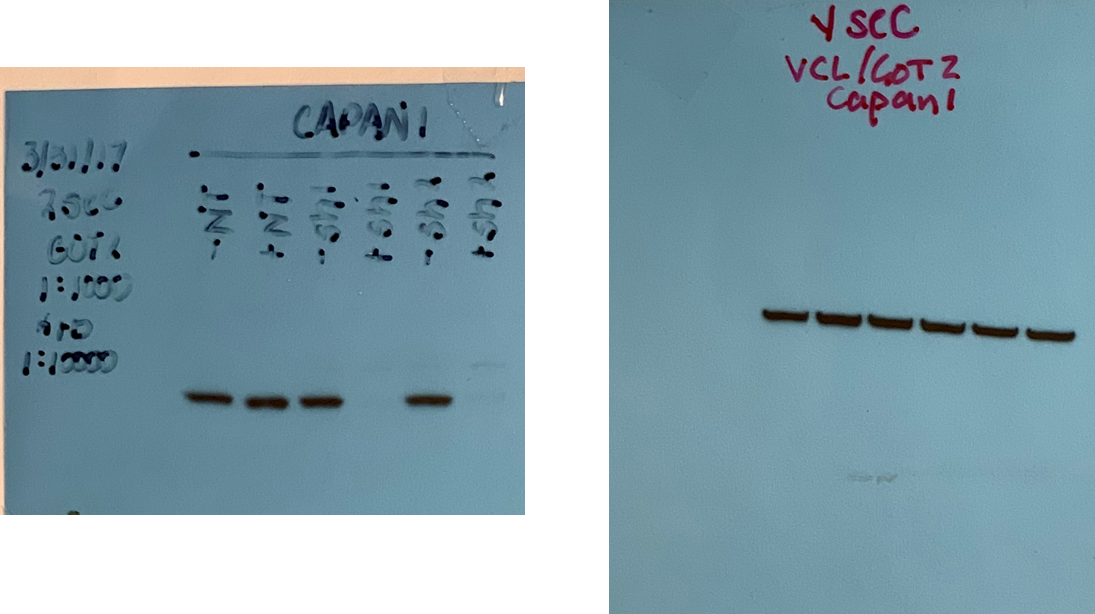


PL45


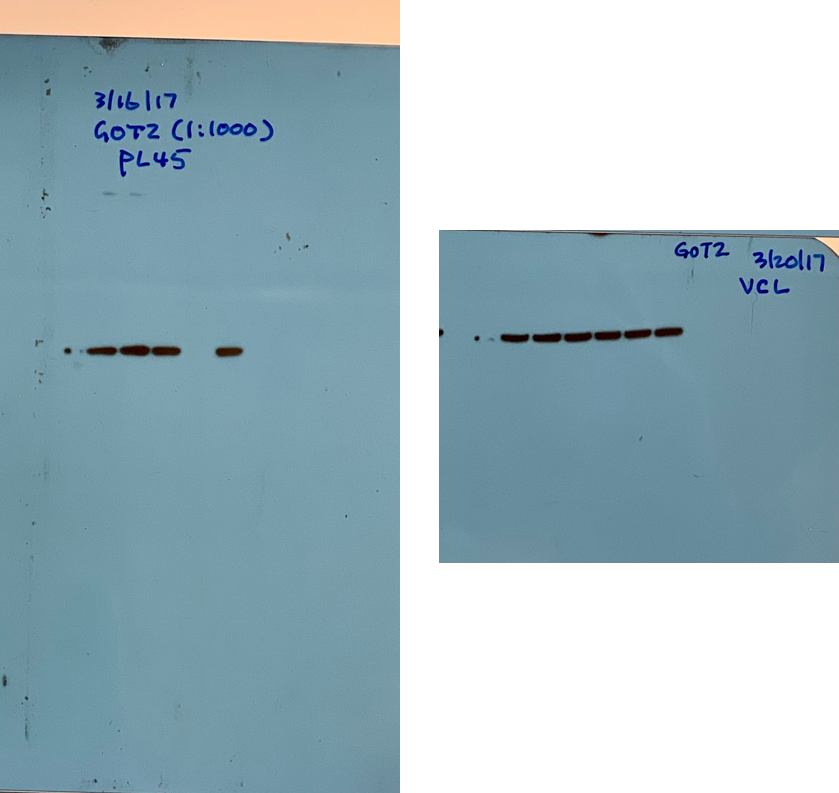


BxPC-3


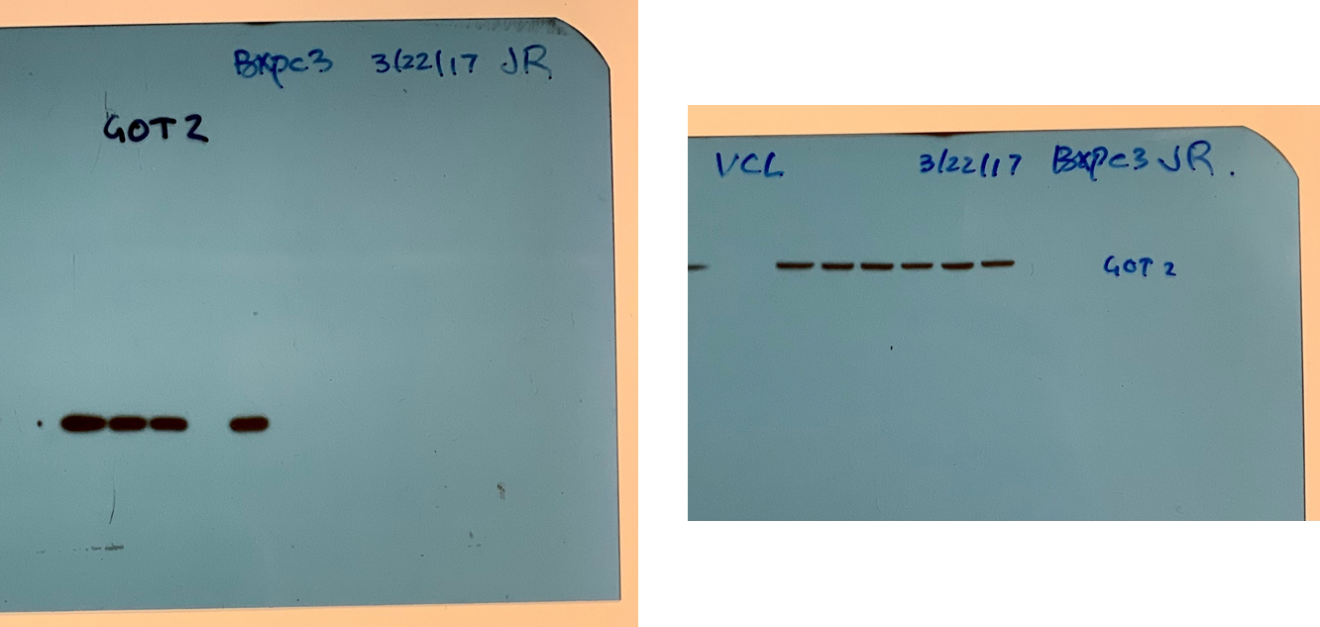


UM76


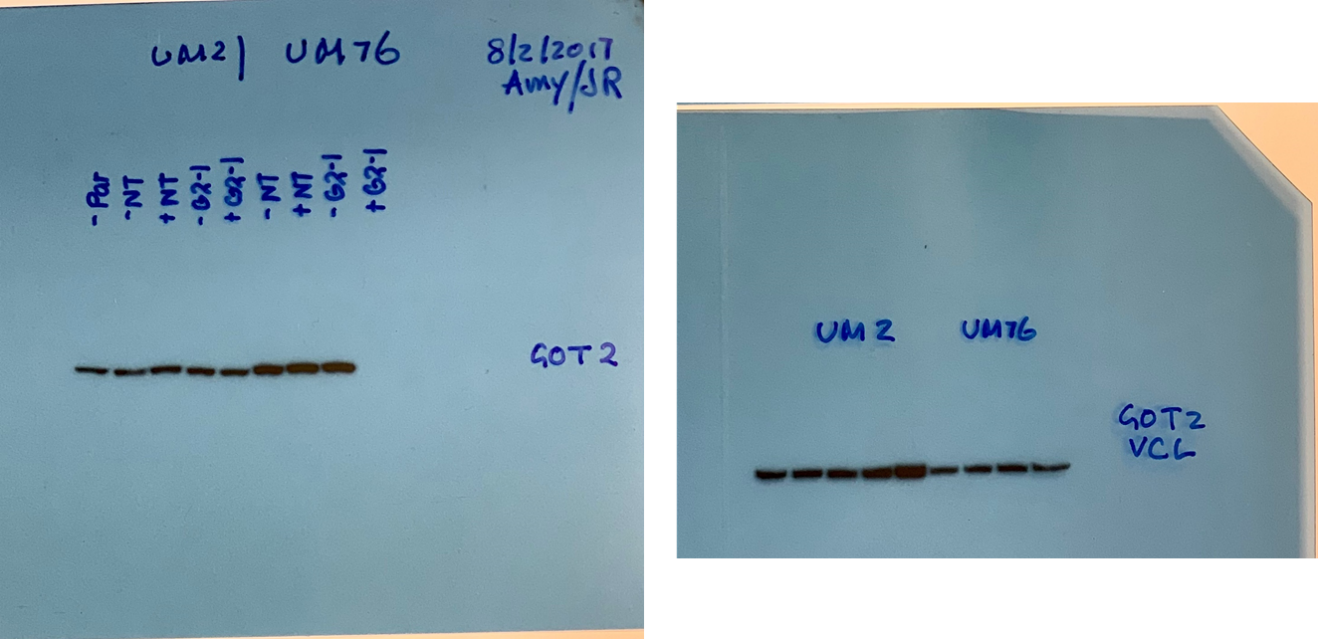


UM19


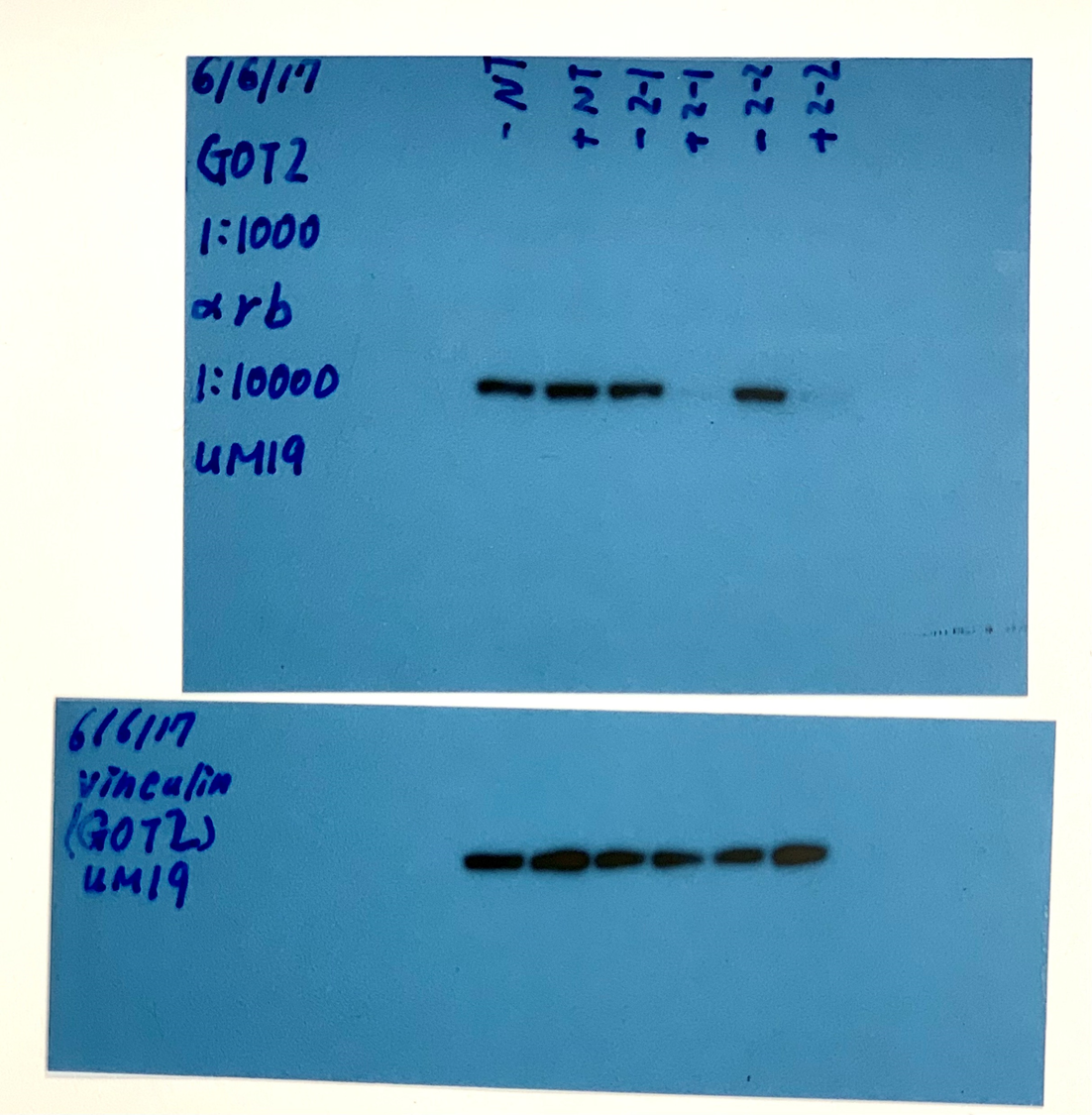


UM6/UM32


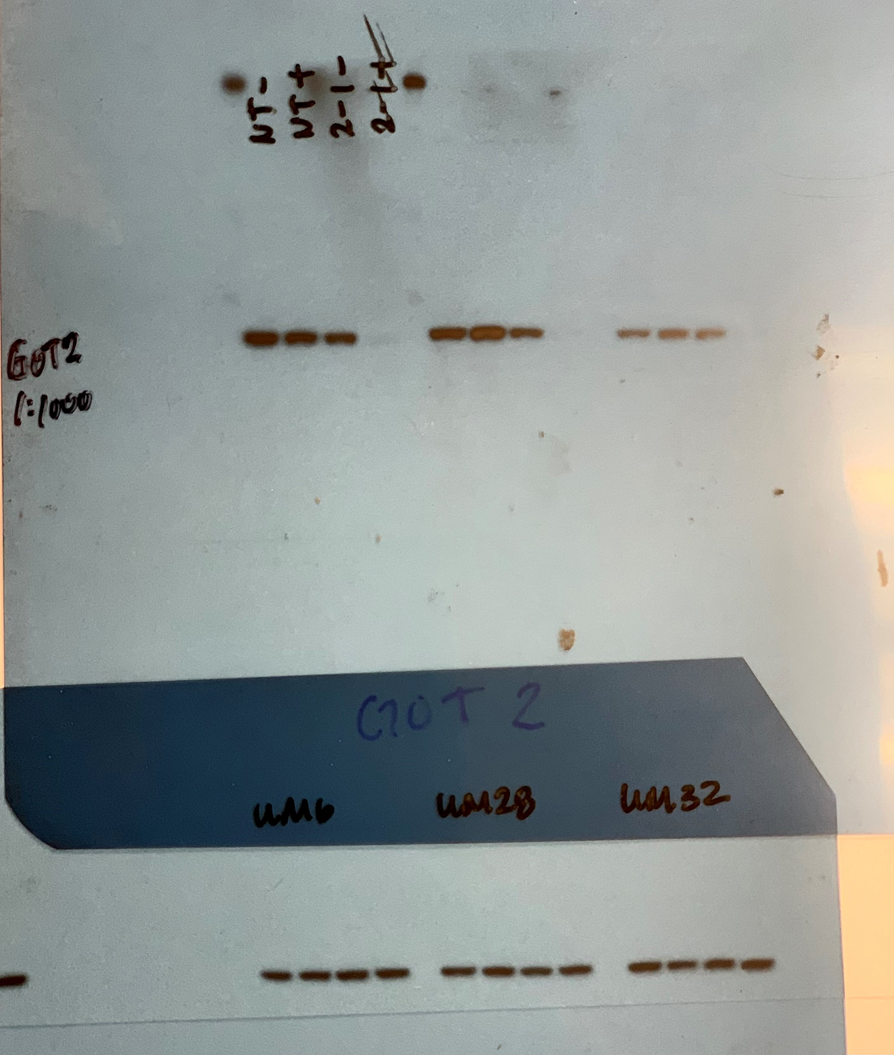


Panc03.27


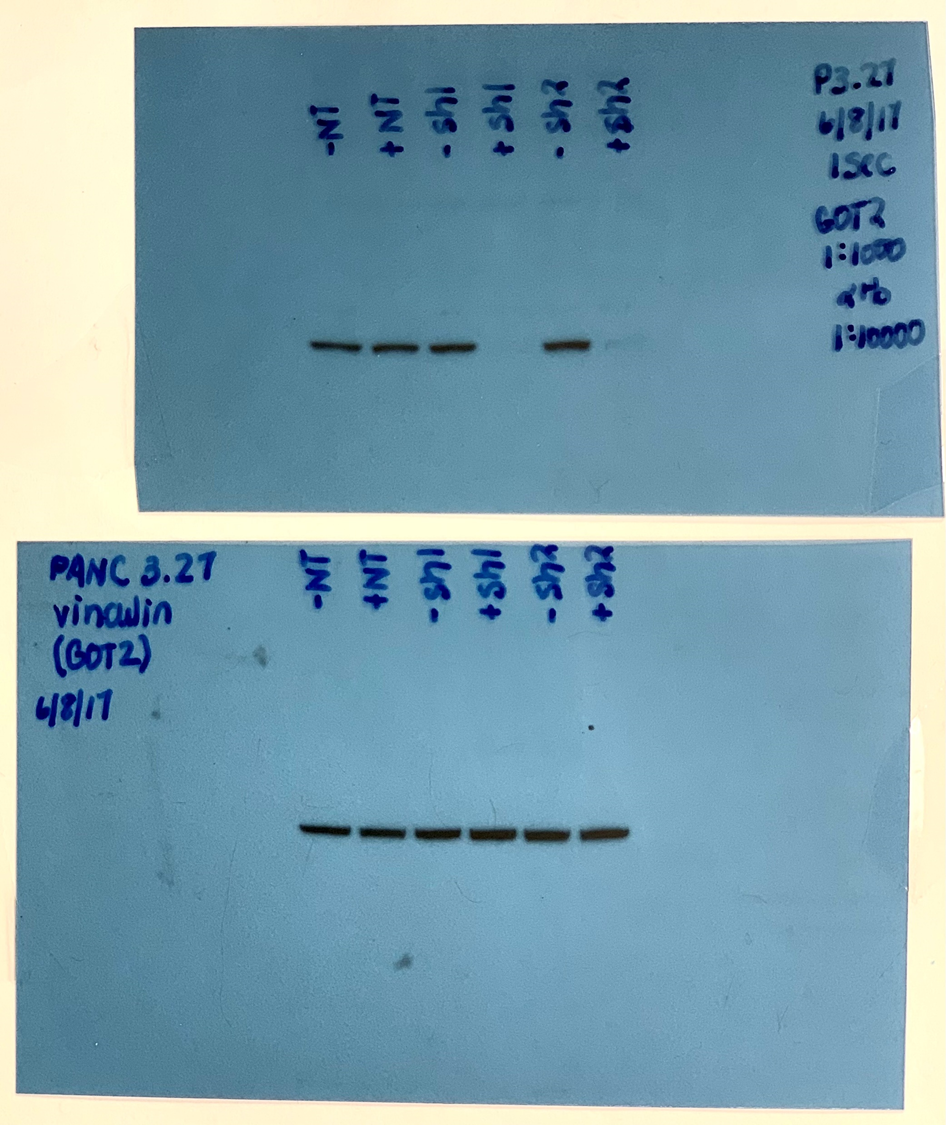


YAPC


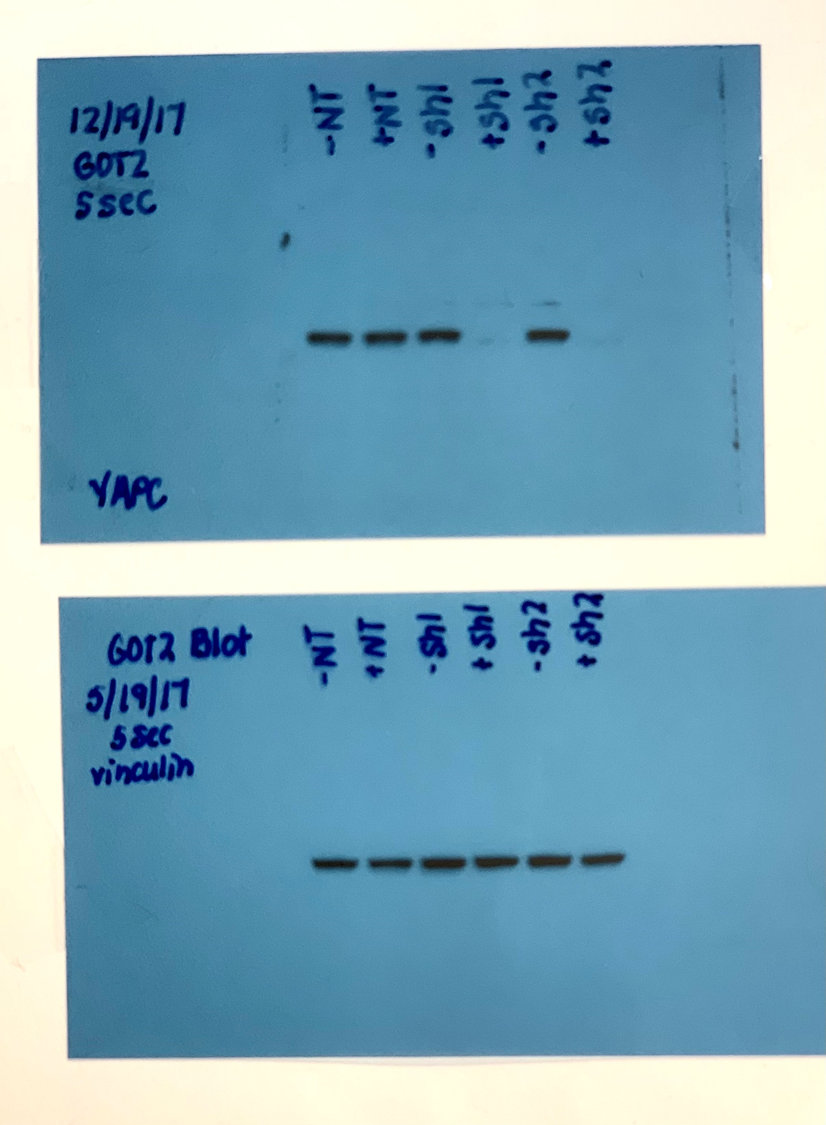


8988T


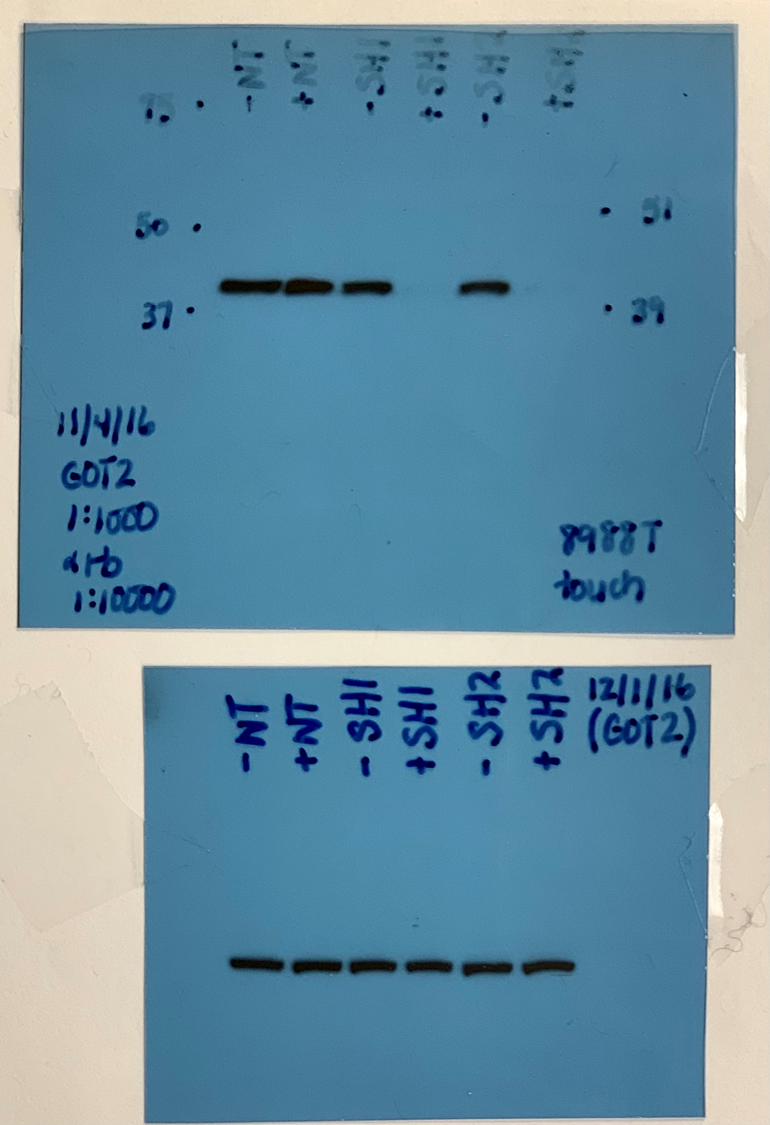


Panc10.05


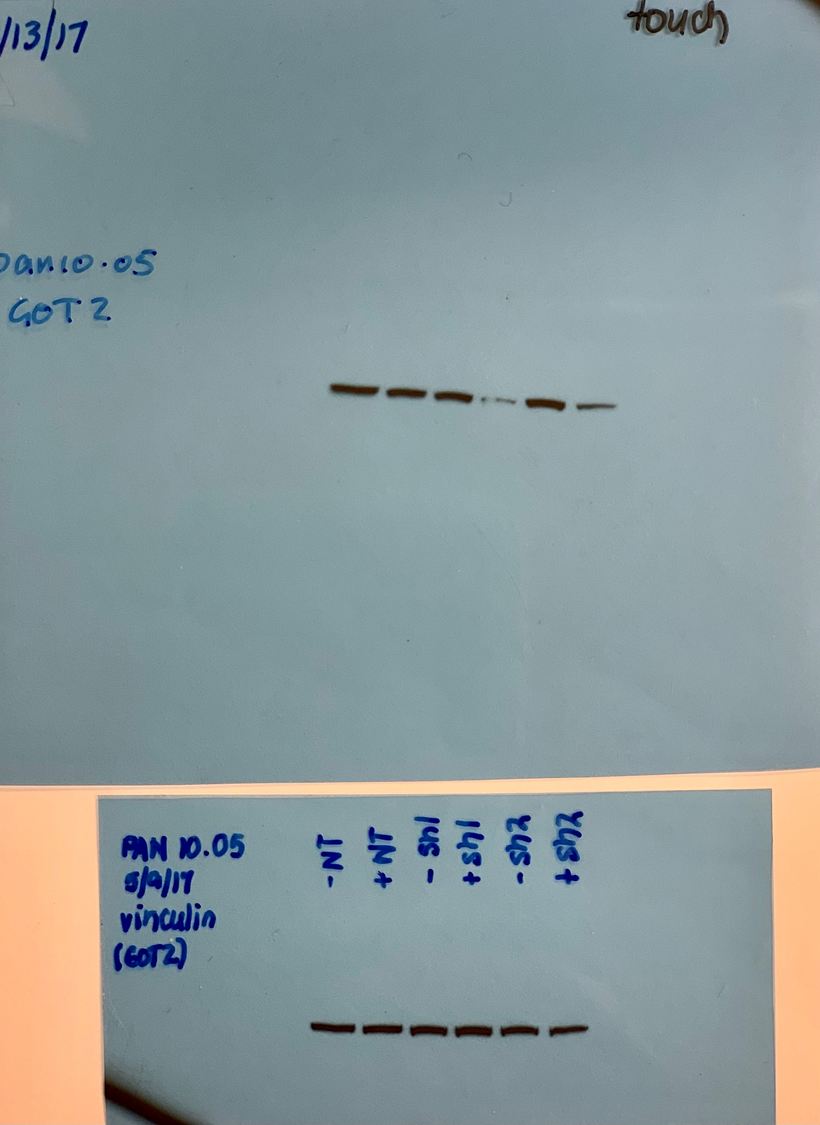


UM53


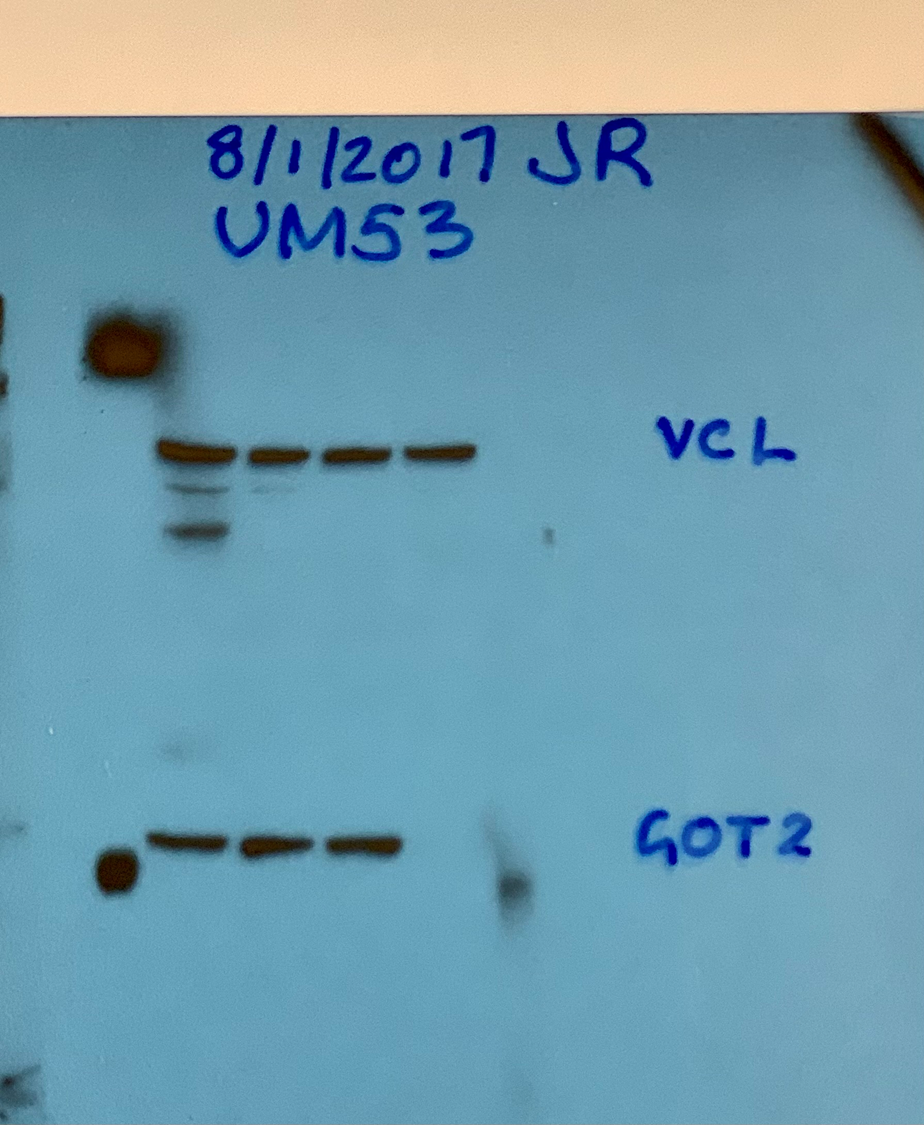


**Figure 1-figure supplement 1B**

Blot1-GOT2, Vinculin loading control


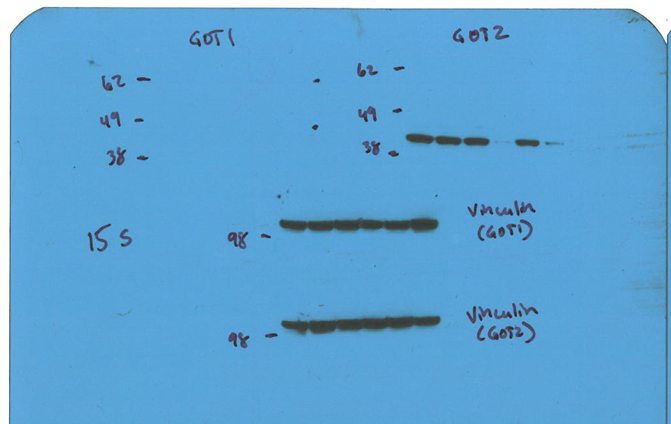


Blot2-GOT1


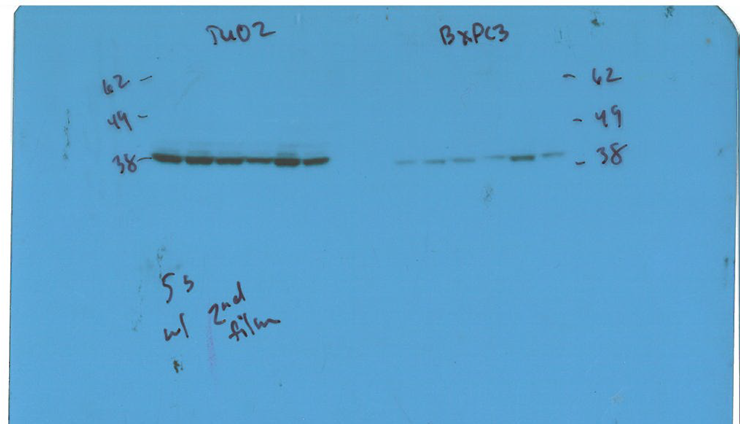


**Figure 1-figure supplement 1E**

hPSC


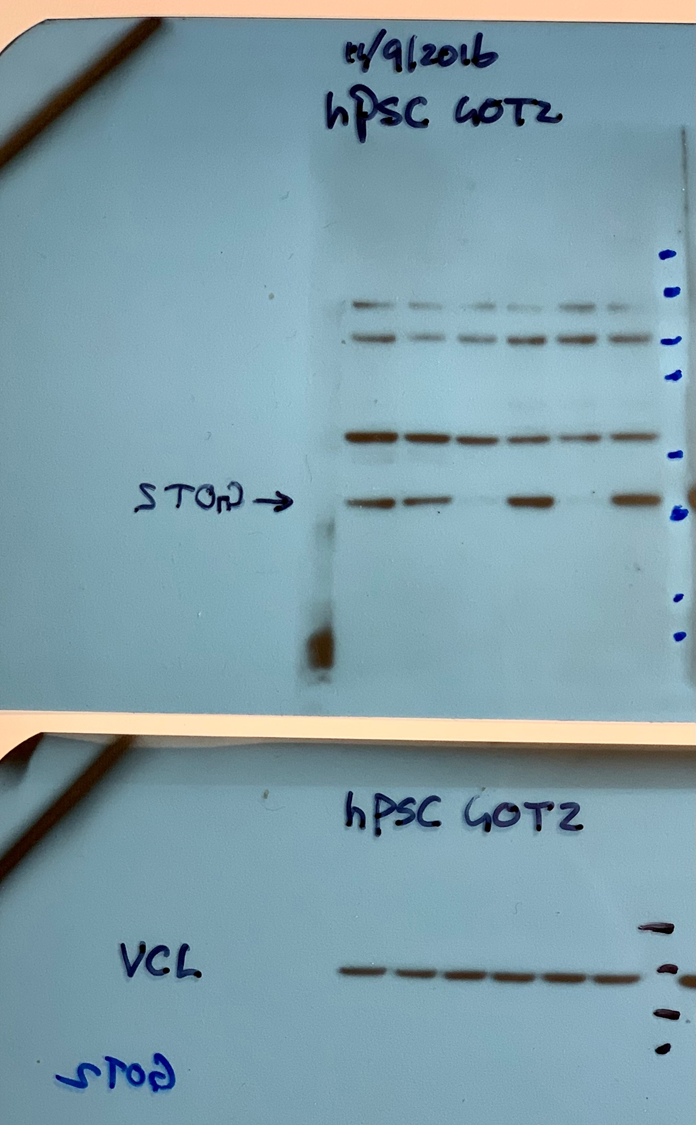


HPNE


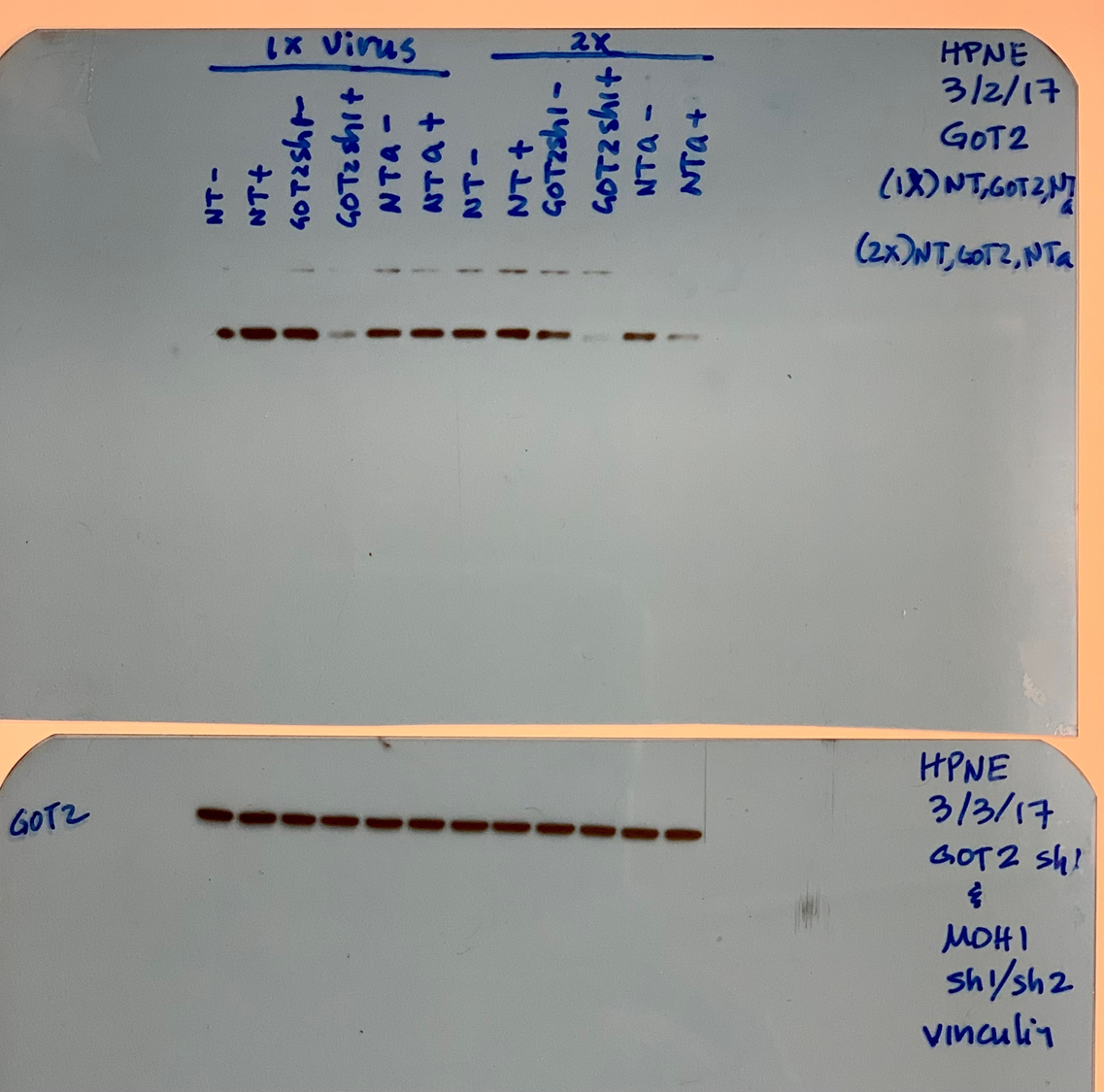

Supplement: Figure 1—figure supplement 1—source data 1. [file elife-73245-fig1-figsupp1-data1.zip › Figure 1-figure supplement 1-source data 1.docx]
