# Supplementary material for: Metabolic requirement for GOT2 in pancreatic cancer depends on environmental context
Source: eLife. 2022 Jul 11;11:e73245. doi: 10.7554/eLife.73245 (PMC9328765; doi:10.7554/eLife.73245)

**Figure 3-figure supplement 1B**

Probe 1-GOT2


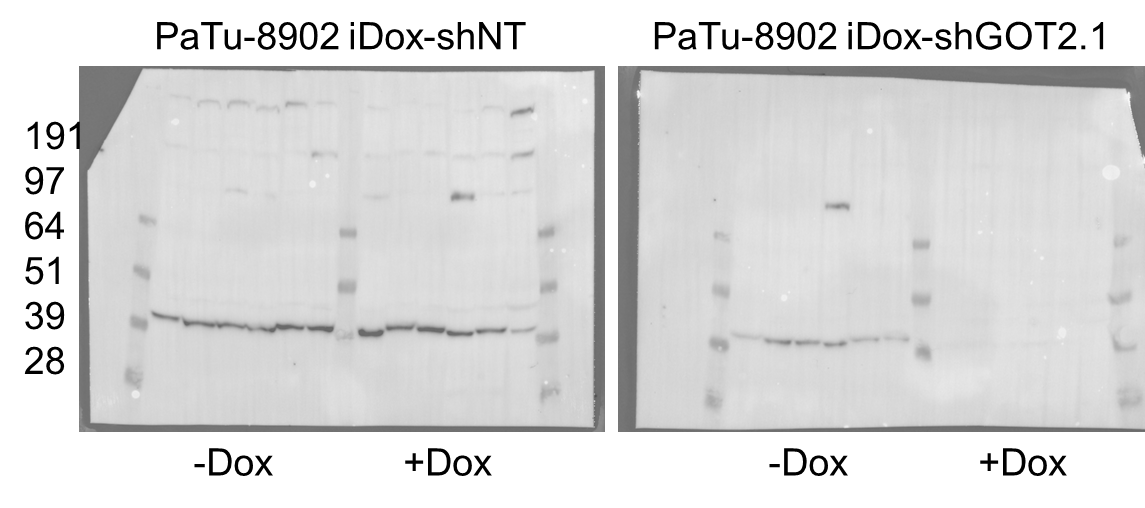


Probe 2-Vinculin loading control


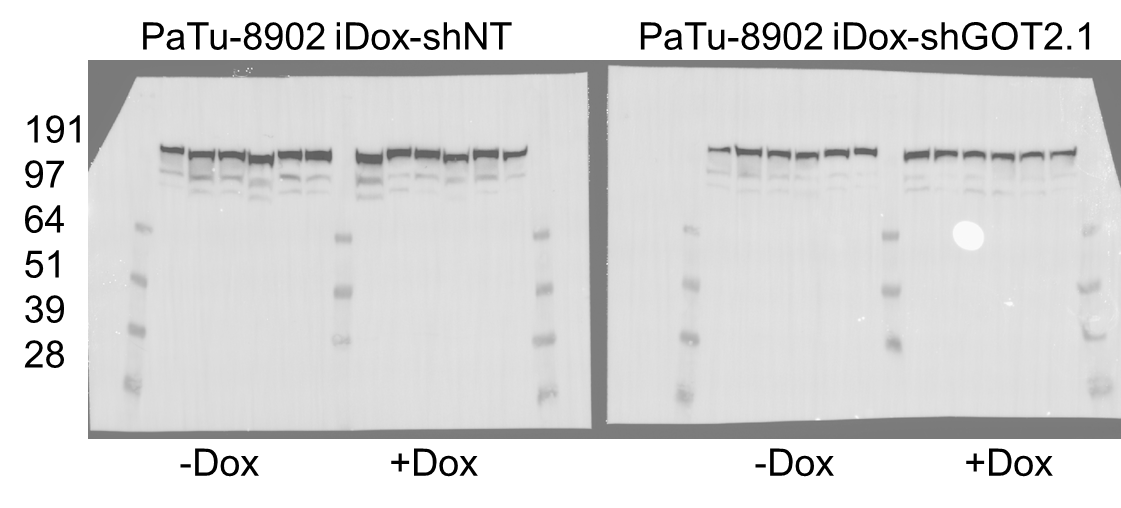


MIAPaCa-2


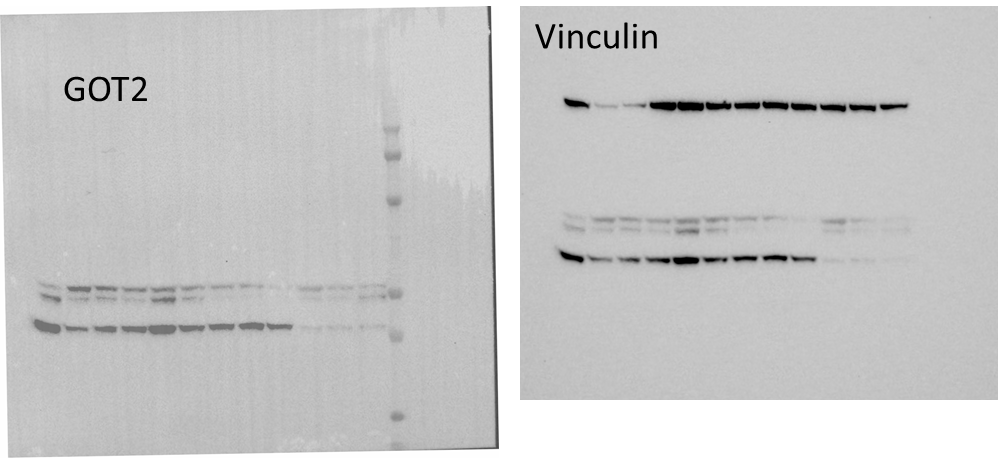


Capan-1


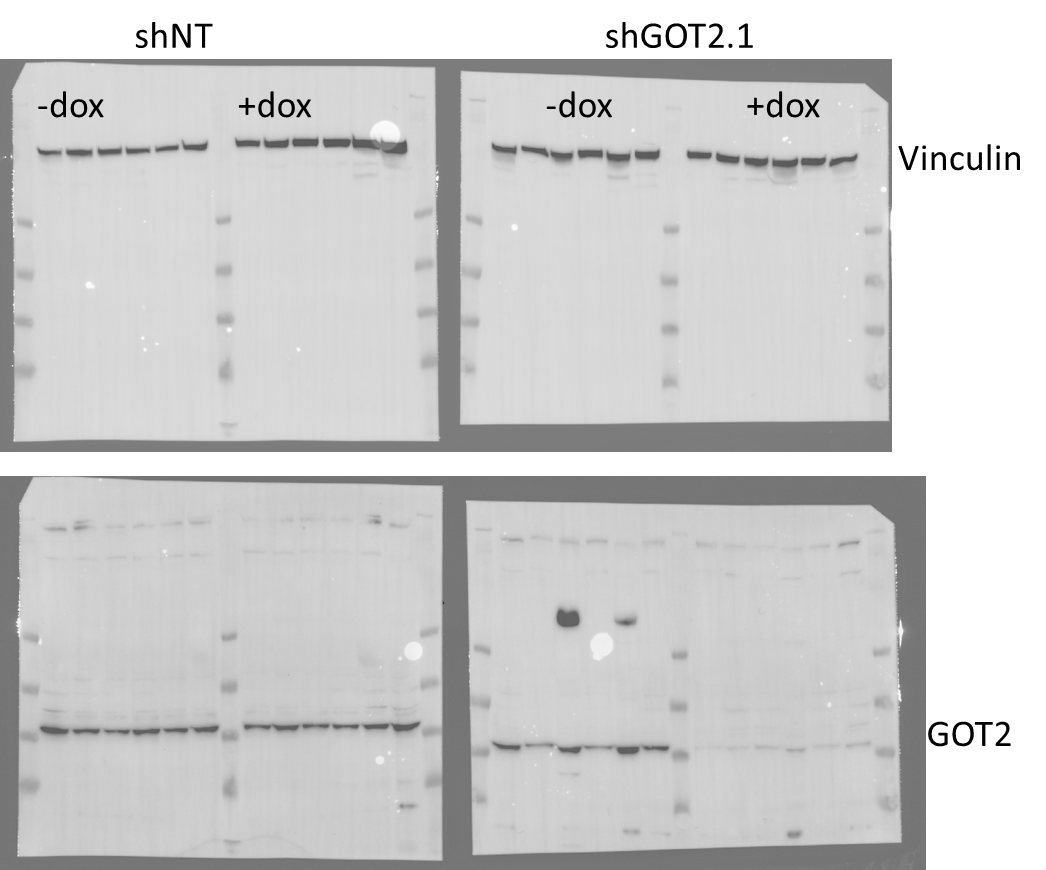


BxPC3


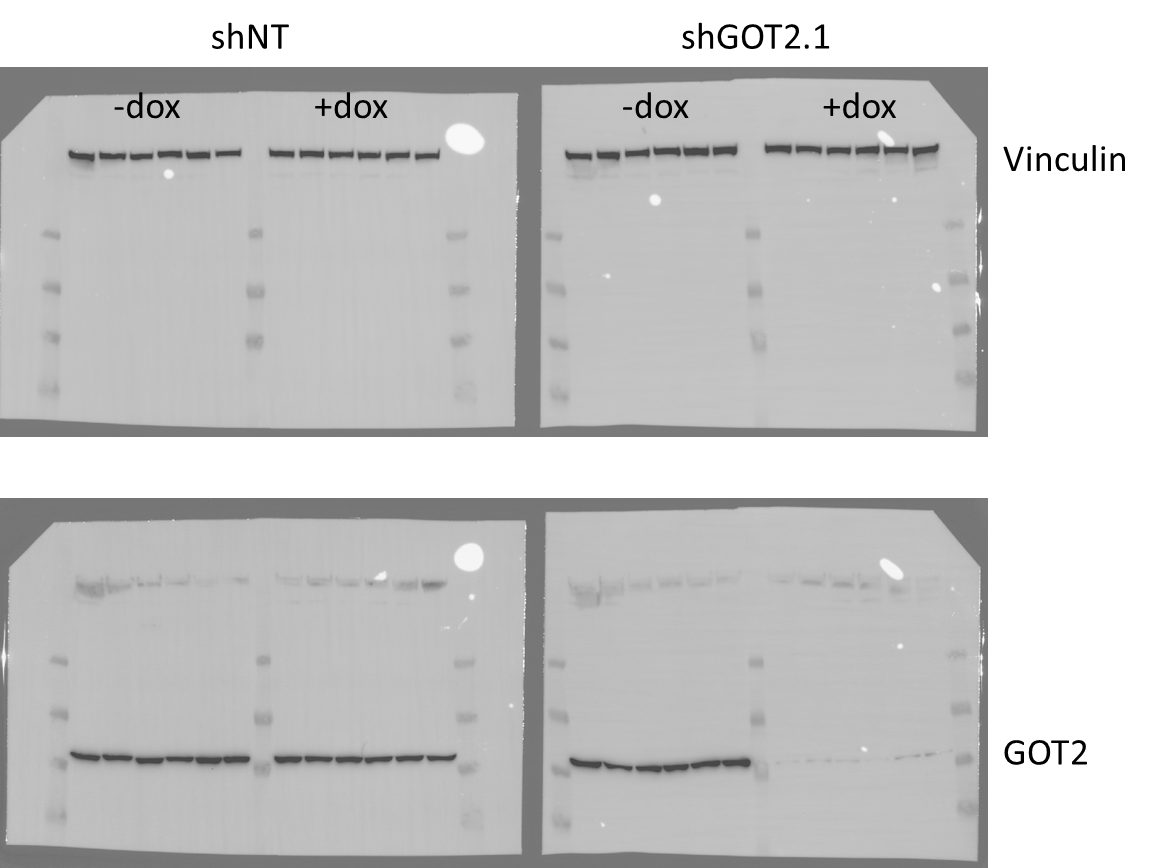


UM53


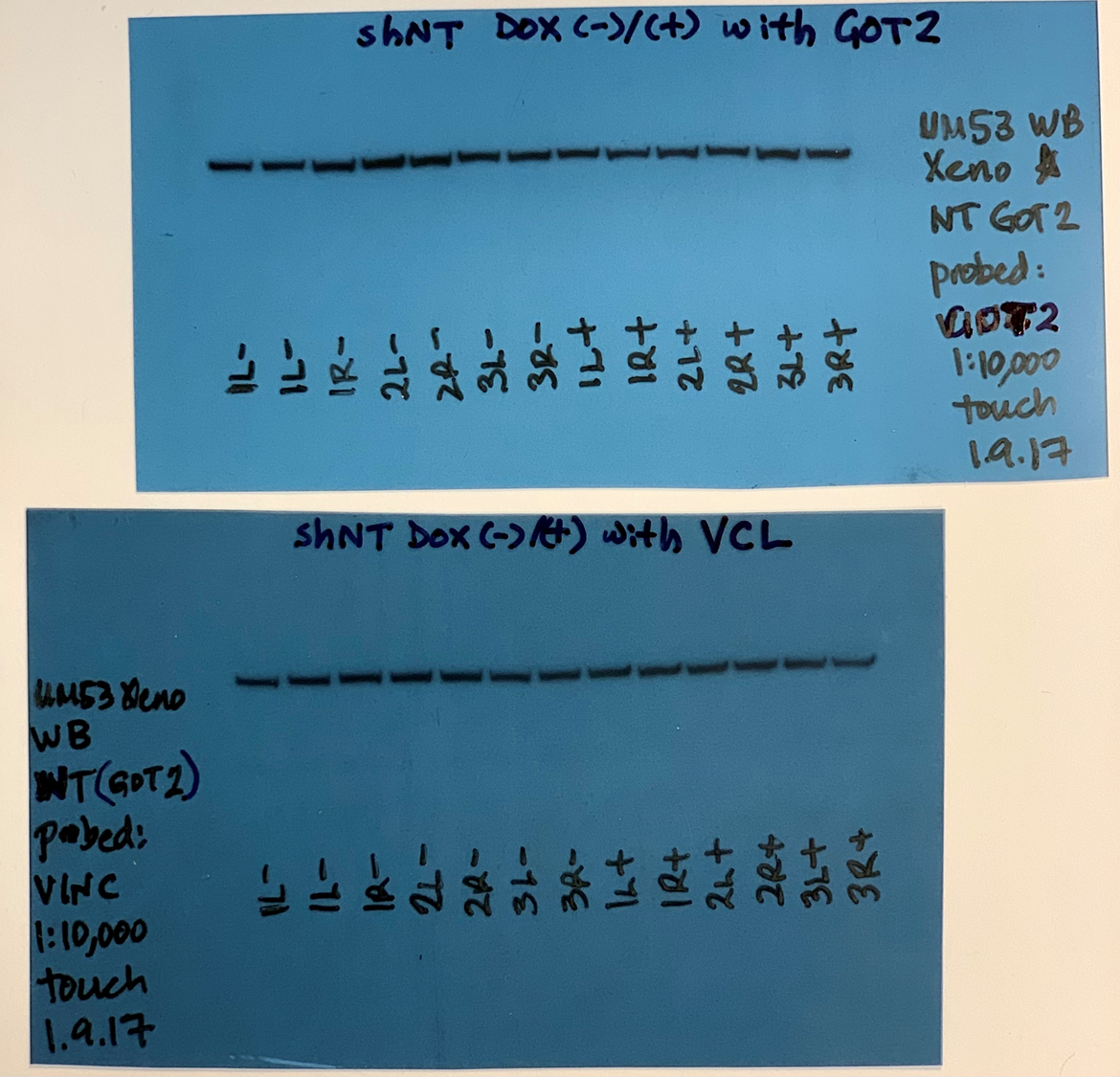


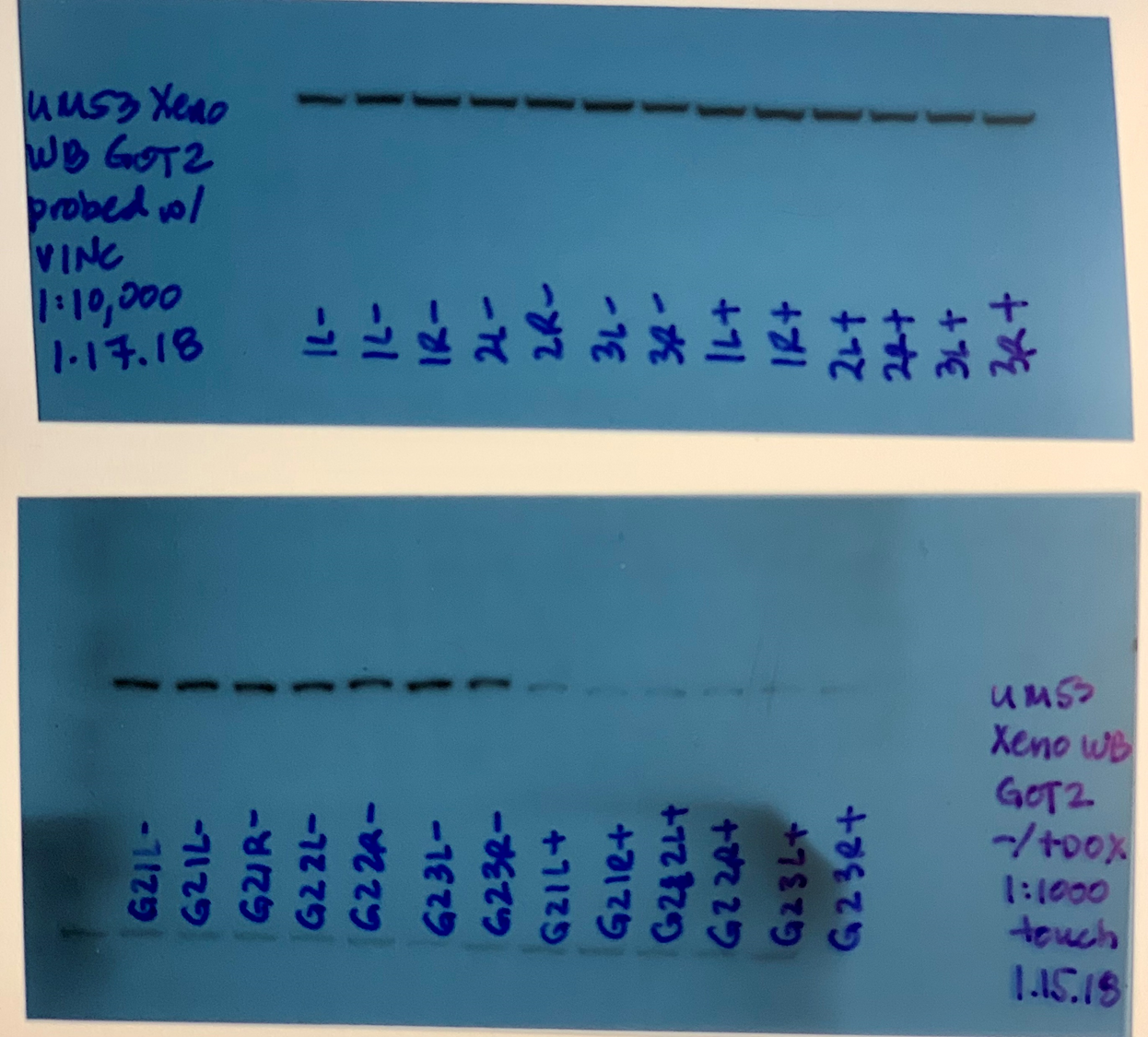


**Figure 3-figure supplement 1G**

Blot 1- GOT1, GOT2, GLUD1, Vinculin loading control


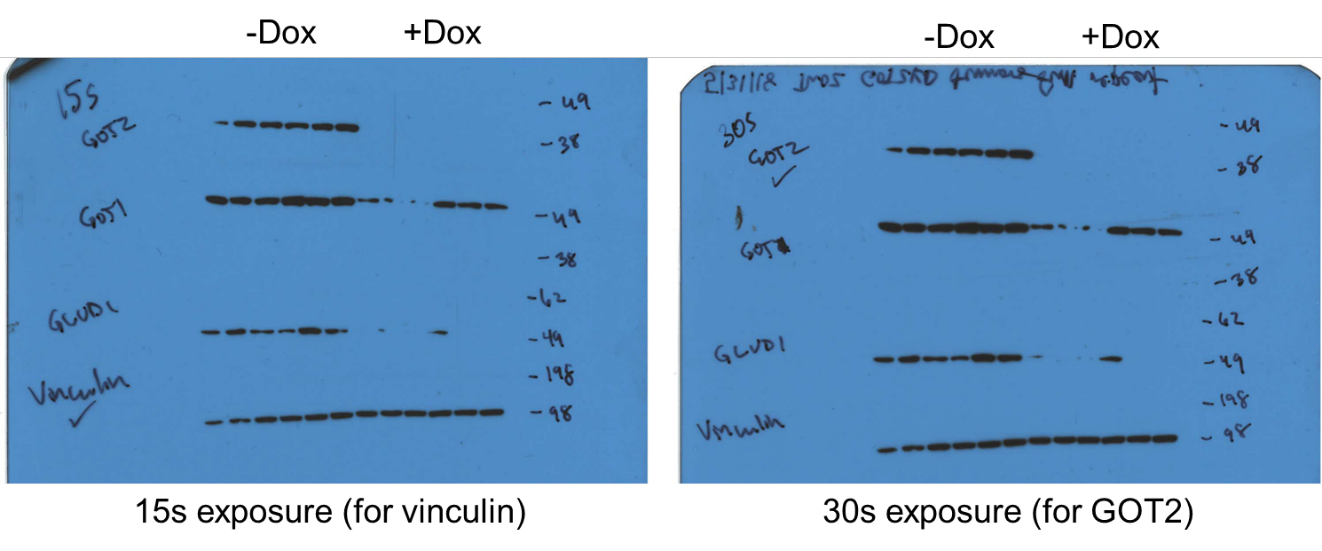


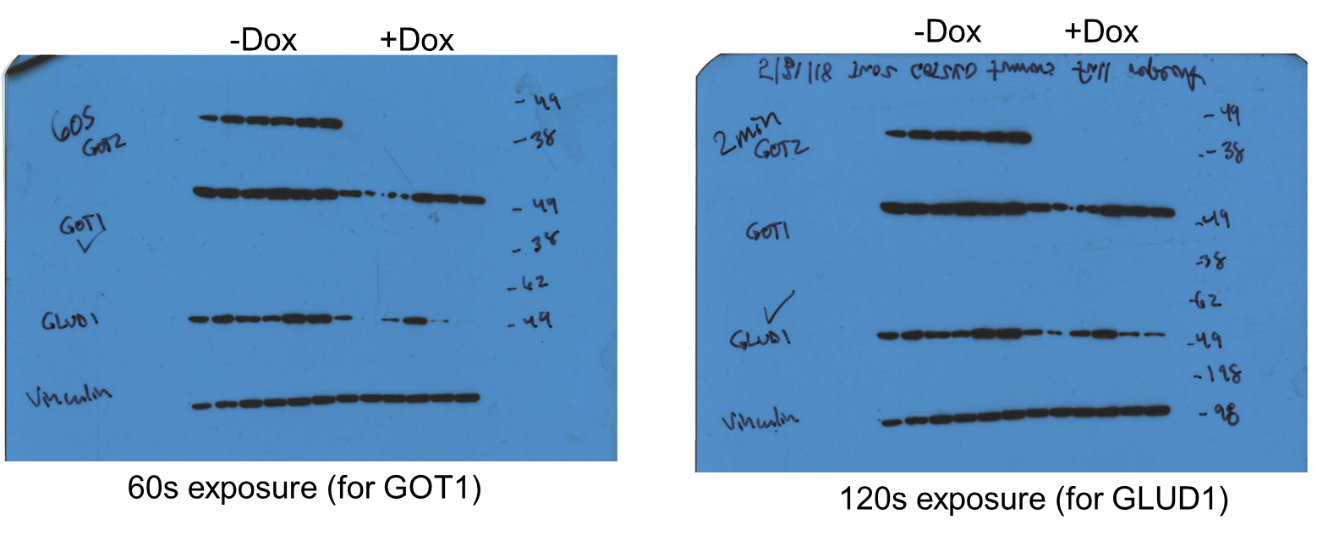


Blot 2-IDH1, vinculin loading control


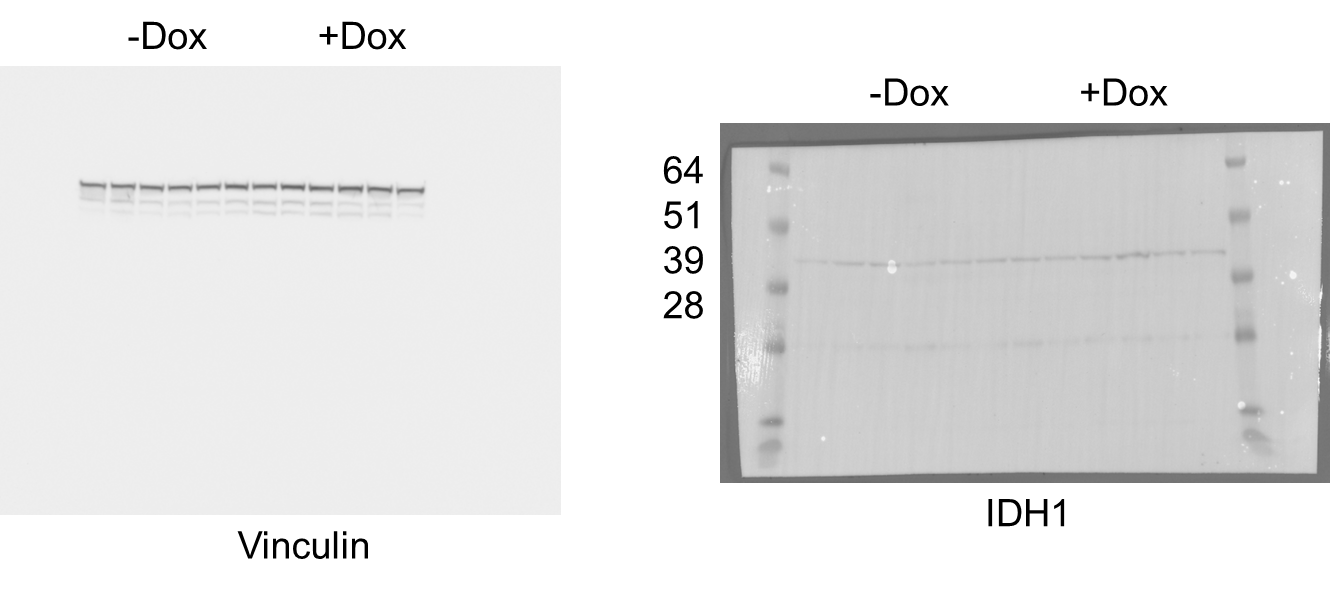

Supplement: Figure 3—figure supplement 1—source data 1. [file elife-73245-fig3-figsupp1-data1.zip › Figure 3-figure supplement 1-source data 1.docx]
