# Supplementary material for: Metabolic requirement for GOT2 in pancreatic cancer depends on environmental context
Source: eLife. 2022 Jul 11;11:e73245. doi: 10.7554/eLife.73245 (PMC9328765; doi:10.7554/eLife.73245)

**Figure 6-figure supplement 2C**


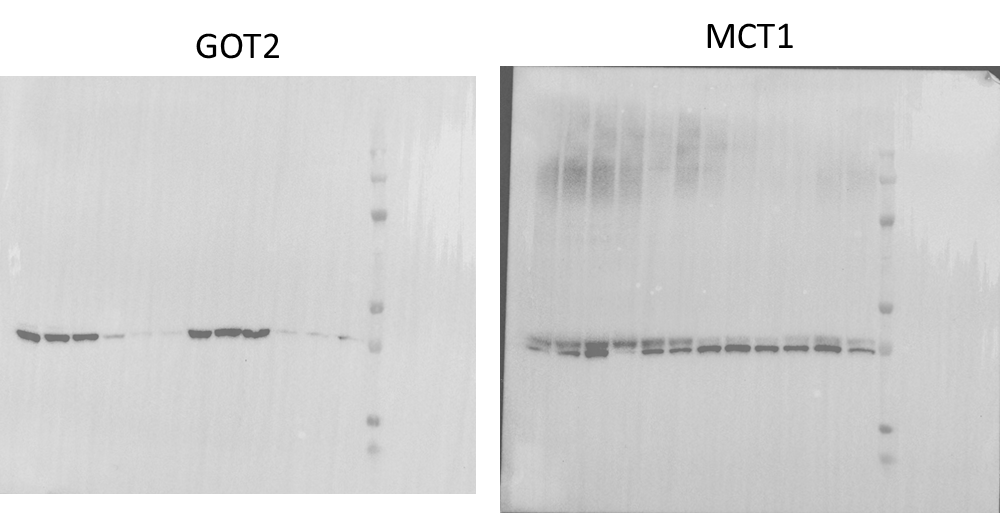


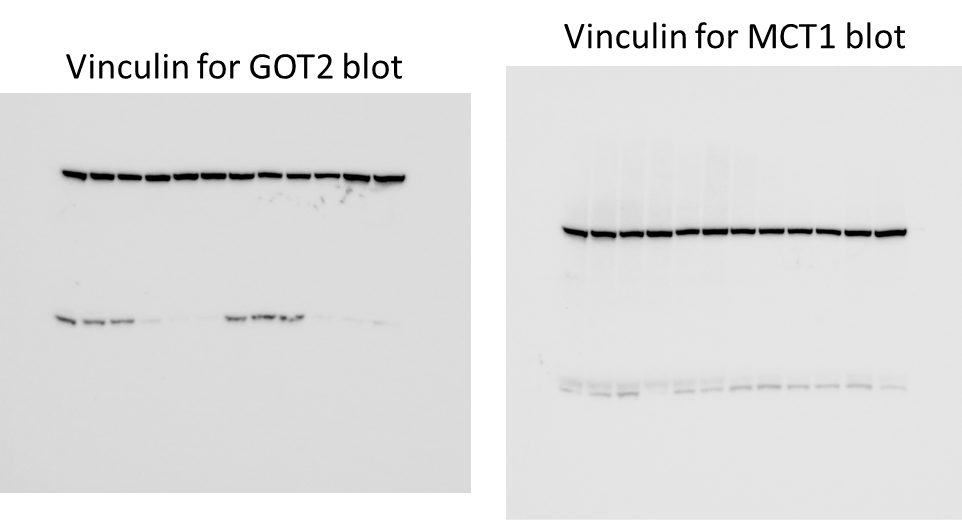


**Figure 6-figure supplement 2D**


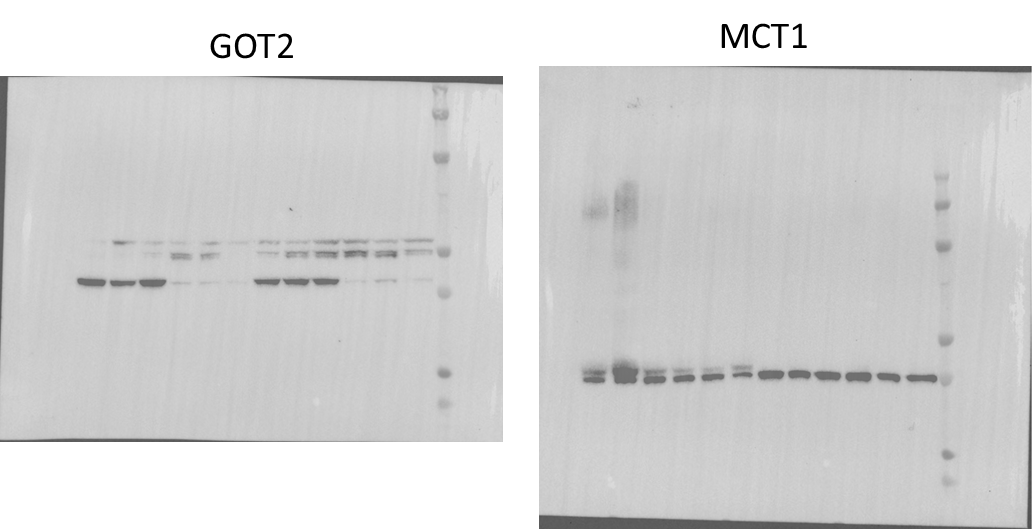


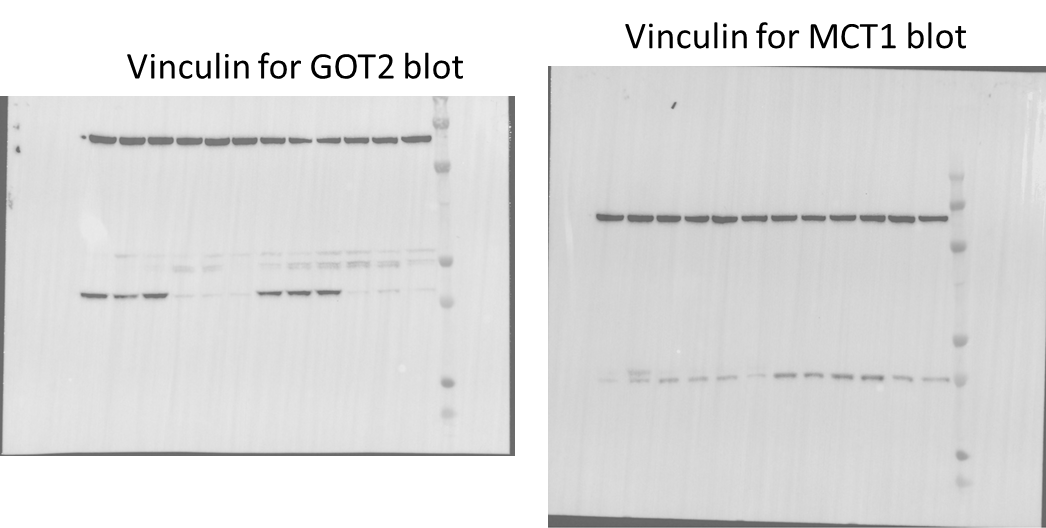


**Figure 6-figure supplement 2F**


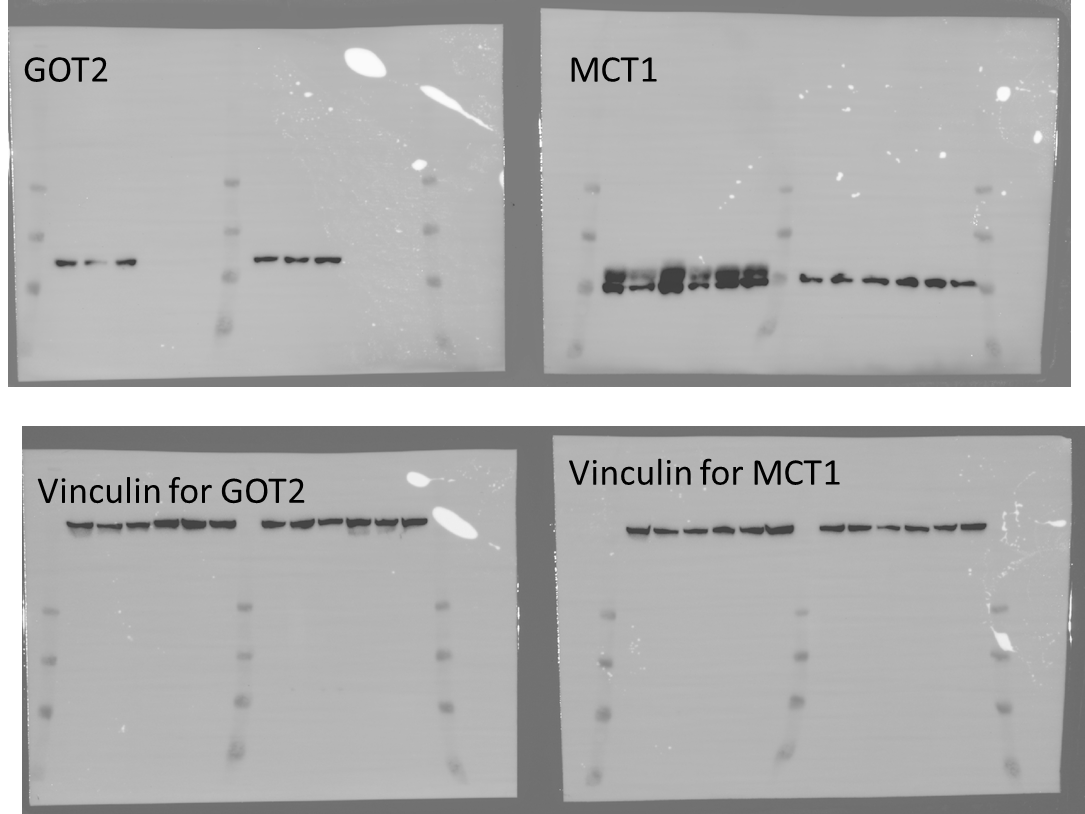


**
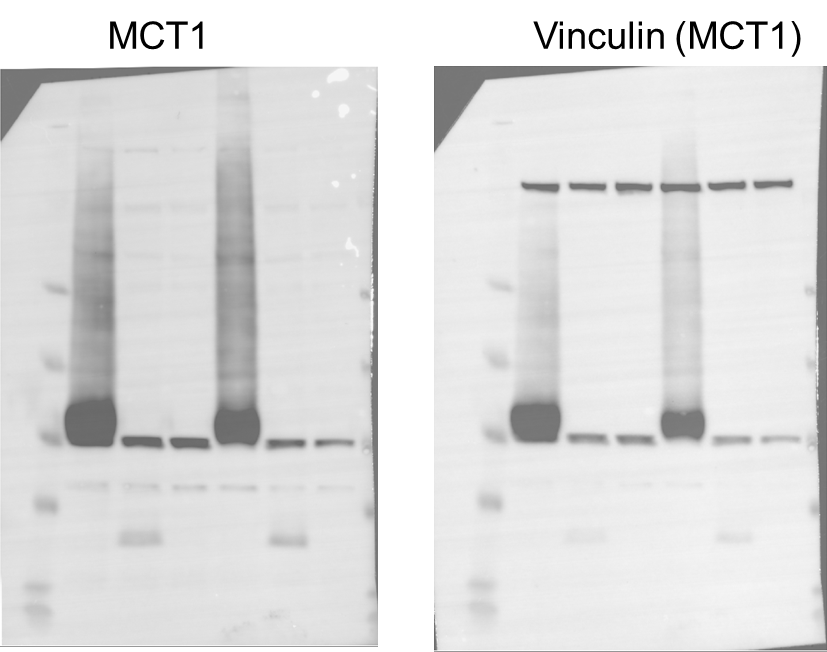

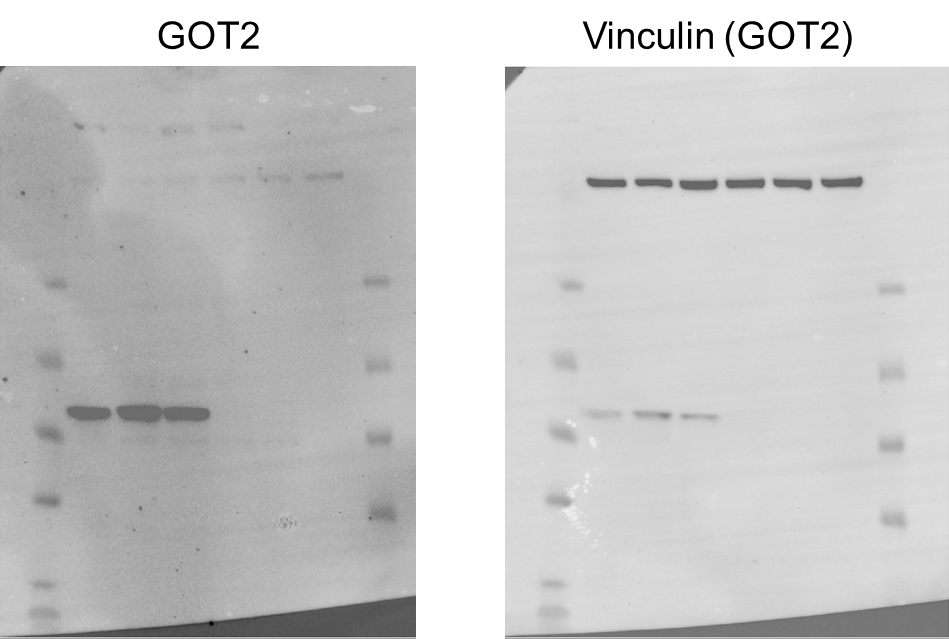
Figure 6-figure supplement 2G**

**
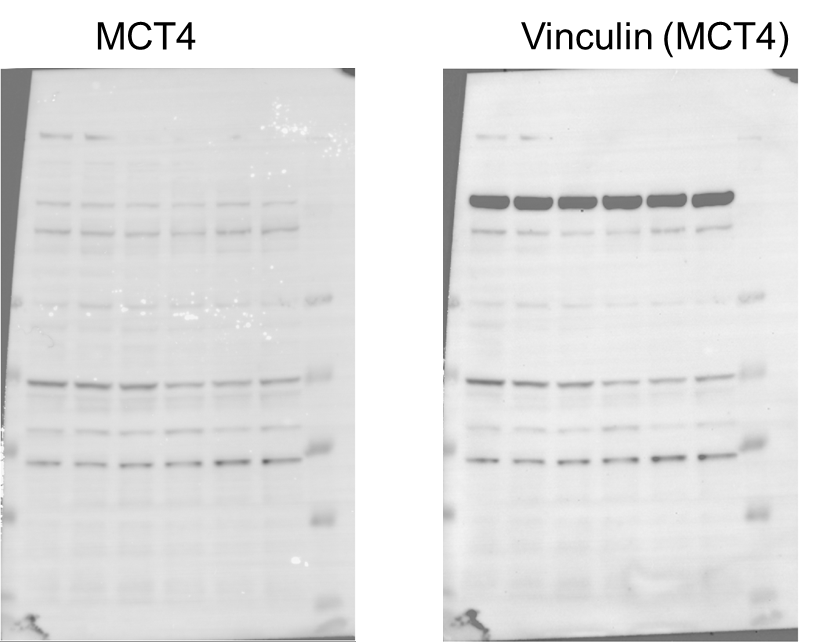
**

**Figure 6-figure supplement 2I**


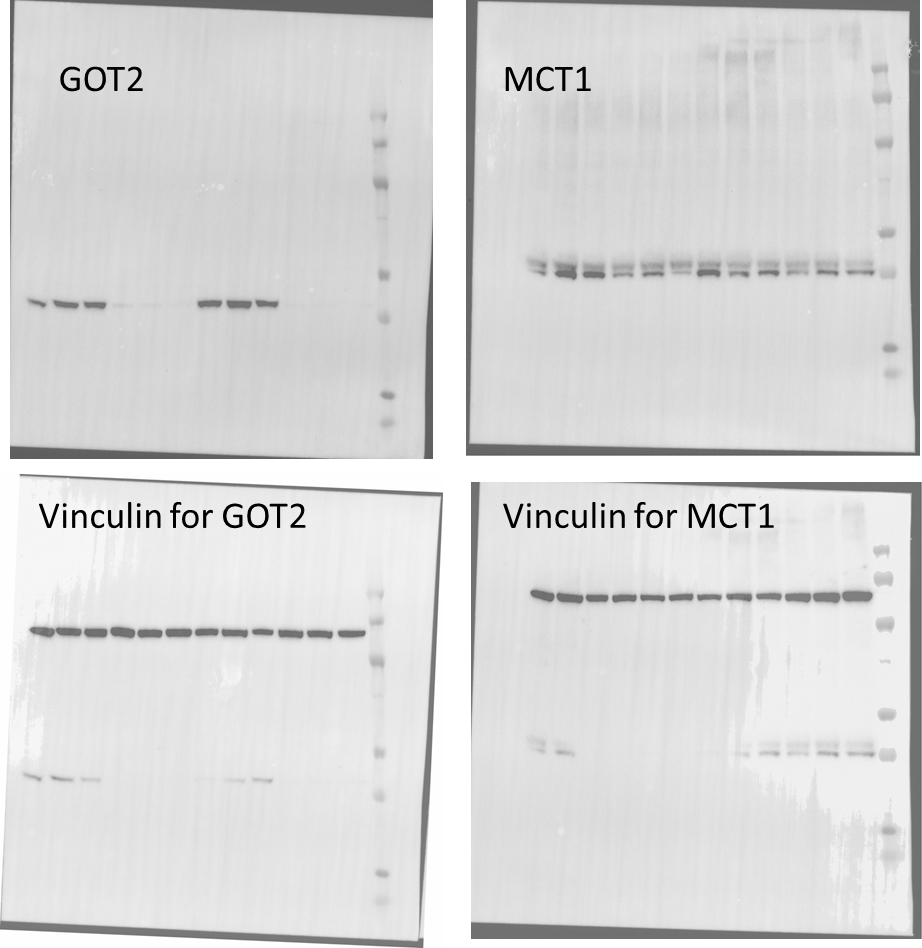

Supplement: Figure 6—figure supplement 2—source data 1. [file elife-73245-fig6-figsupp2-data1.zip › Figure 6-figure supplement 2-source data 1.docx]
